# Clinical diagnosis of metabolic disorders using untargeted metabolomic profiling and disease-specific networks learned from profiling data

**DOI:** 10.1038/s41598-022-10415-5

**Published:** 2022-04-21

**Authors:** Lillian R. Thistlethwaite, Xiqi Li, Lindsay C. Burrage, Kevin Riehle, Joseph G. Hacia, Nancy Braverman, Michael F. Wangler, Marcus J. Miller, Sarah H. Elsea, Aleksandar Milosavljevic

**Affiliations:** 1 Quantitative and Computational Biosciences Program, Baylor College of Medicine, One Baylor Plaza, 400D, Houston, TX 77030 USA; 2 Department of Molecular and Human Genetics, Baylor College of Medicine, Houston, TX USA; 3 Department of Biochemistry and Molecular Medicine, Keck School of Medicine of the University of Southern California, Los Angeles, CA USA; 4 Department of Pediatrics and Human Genetics, McGill University, Montreal, QC Canada; 5 Texas Children’s Hospital, Houston, TX USA; 6 Jan and Dan Duncan Texas Children’s Hospital Neurological Research Institute, Houston, TX USA; 7 Department of Medical and Molecular Genetics, Indiana University School of Medicine, Indianapolis, IN USA

**Keywords:** Machine learning, Metabolomics, Diagnostic markers

## Abstract

Untargeted metabolomics is a global molecular profiling technology that can be used to screen for inborn errors of metabolism (IEMs). Metabolite perturbations are evaluated based on current knowledge of specific metabolic pathway deficiencies, a manual diagnostic process that is qualitative, has limited scalability, and is not equipped to learn from accumulating clinical data. Our purpose was to improve upon manual diagnosis of IEMs in the clinic by developing novel computational methods for analyzing untargeted metabolomics data. We employed CTD, an automated computational diagnostic method that “**c**onnects **t**he **d**ots” between metabolite perturbations observed in individual metabolomics profiling data and modules identified in disease­specific metabolite co-perturbation networks learned from prior profiling data. We also extended CTD to calculate distances between any two individuals (CTDncd) and between an individual and a disease state (CTDdm), to provide additional network-quantified predictors for use in diagnosis. We show that across 539 plasma samples, CTD-based network-quantified measures can reproduce accurate diagnosis of 16 different IEMs, including adenylosuccinase deficiency, argininemia, argininosuccinic aciduria, aromatic l-amino acid decarboxylase deficiency, cerebral creatine deficiency syndrome type 2, citrullinemia, cobalamin biosynthesis defect, GABA-transaminase deficiency, glutaric acidemia type 1, maple syrup urine disease, methylmalonic aciduria, ornithine transcarbamylase deficiency, phenylketonuria, propionic acidemia, rhizomelic chondrodysplasia punctata, and the Zellweger spectrum disorders. Our approach can be used to supplement information from biochemical pathways and has the potential to significantly enhance the interpretation of variants of uncertain significance uncovered by exome sequencing. CTD, CTDdm, and CTDncd can serve as an essential toolset for biological interpretation of untargeted metabolomics data that overcomes limitations associated with manual diagnosis to assist diagnosticians in clinical decision-making. By automating and quantifying the interpretation of perturbation patterns, CTD can improve the speed and confidence by which clinical laboratory directors make diagnostic and treatment decisions, while automatically improving performance with new case data.

## Introduction

While the adoption of exome sequencing in the clinic brought major improvements in diagnostic accuracy and speed, it has also brought with it the challenge of interpreting numerous variants of uncertain significance (VUSs). A wide variety of diagnostic approaches have been developed to connect observed genomic alterations with observed clinical phenotypes. For example, initiatives such as the Matchmaker Exchange^1^ use clinical descriptions and semantic similarity metrics to calculate similarity between individuals^2^. A “match” between individuals with highly conspicuous clinical phenotypes can significantly improve the power to find causative genetic variants in cases of Mendelian disease. Unfortunately, many clinical phenotypes observed in individuals with inborn errors of metabolism (IEMs) are non-specific (e.g., seizures, intellectual disability, diarrhea, vomiting, and poor feeding), making diagnosis based solely on clinical descriptions difficult.

In contrast to clinical phenotypes, metabolic defects observed in many IEMs cause highly distinct metabolite perturbation patterns in plasma, represented by abnormal accumulation or depletion of essential metabolites stemming from an affected protein that has enzymatic, carrier, receptor, or structural roles in cellular metabolism. As a result, metabolomics can help bridge existing knowledge gaps between causal genetic variation and observed clinical phenotypes.

Functional evidence from patient-derived “omic” data (e.g., the transcriptome, proteome, and metabolome) is recognized as one of the key factors in resolving VUSs. The widely adopted American College of Medical Genetics and the Association for Molecular Pathology (ACMG/AMP) Guidelines^3^ define evidence category PS3, which provides means for formally incorporating functional evidence from “well-established” functional studies^3^. Untargeted clinical testing metabolomics^4^ is a functional diagnostic test which has allowed for the successful diagnosis of many cases of metabolic disorders that would be hard to diagnose using clinical phenotype descriptions and targeted tests alone^5–10^. Nevertheless, wide application of this source of evidence requires a quantitative, transparent, and computationally efficient method for detecting and comparing disease-specific multi-metabolite perturbations. Various automated tools for predicting the pathogenicity of genetic variants have been developed (e.g., SIFT, PolyPhen2, CADD, DANN)^11–14^, but none incorporate metabolomic profiling, nor other types of precise molecular phenotyping information.

Many computational methods^15–18^ have been developed for the analysis of clinical research metabolomics data. Unlike clinical research metabolomics, which follows a case–control observational study design and relies on population-based statistical power, clinical metabolomics testing facilitates the interpretation of an individual (N-of-1) case in relation to a reference population of healthy controls^4,19^. Other than the manual inspection of untargeted metabolomics data currently used to diagnose individual cases, few alternative methods are suitable for interpreting multi-metabolite perturbations observed in N-of-1 cases, and of these available alternatives, many rely on knowledge-driven modelling (e.g., pathway maps and biomarker lists) approaches^20^.

The CTD method^21^ is a novel information-theoretic method that assigns statistical significance to sets of metabolites based on their connectedness in disease-specific metabolite “co-perturbation” networks derived from accumulating patient data. A network contains metabolite nodes, and the weighted edges connect metabolites that are co-perturbed in a specific disease. Gaussian graphical models are used to compute edge weights, which indicate the strength of positive or negative partial correlation between metabolites. Given a disease-specific network and a set of metabolites that are perturbed in a given individual, CTD identifies a subset of perturbed metabolites that are highly connected within the network. The CTD method uses an efficient algorithm that can handle highly dense (“hairball”) networks and outputs small p values for highly connected metabolite sets and large p values for sparsely connected metabolites sets. Unlike any other method of similar complexity, CTD does not require computationally costly permutation testing to establish p values of combinatorial patterns of multi-metabolite perturbations and can thus be used to interpret untargeted metabolomic profiles of individual patients in an efficient and rigorous way.

To interpret perturbations observed in N-of-1 metabolomics profiles without relying just on prior biochemical pathway knowledge, we applied the CTD method^21^ to existing and newly acquired datasets and introduce the CTDdm and CTDncd distance methods, both extensions of CTD, to serve as additional predictors for diagnosis. We assessed if CTD-based network-quantified measures could reproduce accurate diagnosis of IEMs and whether these measures hold long-term value to supplement existing information from biochemical pathways in order to assist in interpreting VUSs. We provide evidence that CTD-based metrics can indeed expedite the analysis of complex metabolomic datasets and increase the sensitivity of clinical diagnostic pipelines for clinical purposes that include identifying precision treatments for individuals with IEMs.

## Methods

### Data collection

**Figure 1:**
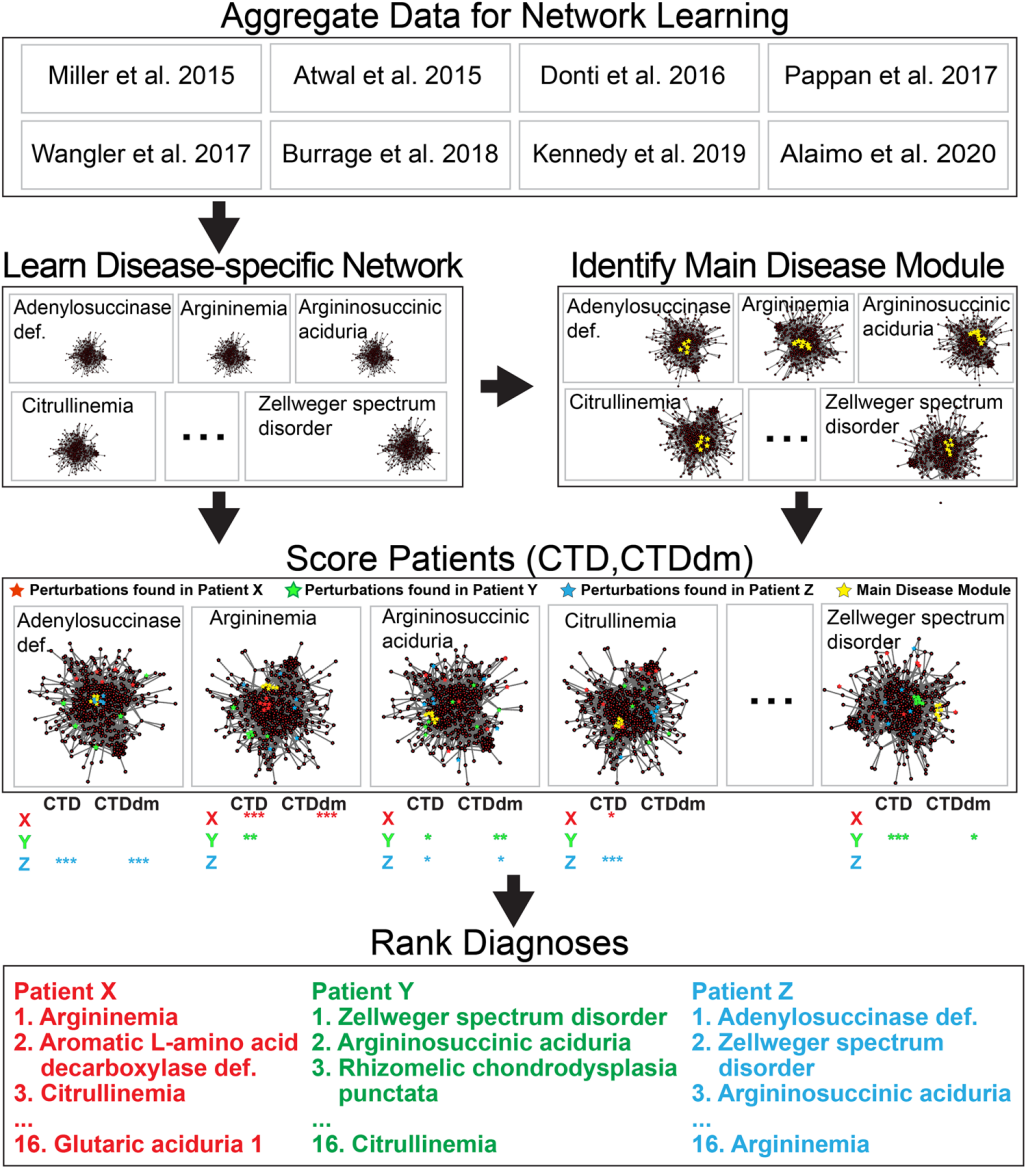
Overview of CTD-based data-driven diagnostic approach.

Data used represent a meta-analysis of untargeted metabolomics plasma samples collected from previously reported studies^5–10,22,23^, as well as previously unreported samples (Table 1, Fig. 1). All study procedures were approved by the Institutional Review Board (IRB) of the Baylor College of Medicine and complied with all relevant guidelines and regulations. For some of the studies, informed consent was obtained and for others, it was waived by the Baylor College of Medicine’s IRB-approved waiver of informed consent. All sample data were de-identified. While all sample data were processed similarly^24^, some data differ in sample source (e.g., heparin vs. EDTA plasma) and platform specifications (e.g., mass analyzer) (Table 1). Sample data were generated by Baylor Genetics in collaboration with Metabolon, Inc. (Morrisville, NC) on referred clinical or research samples. Twenty-one research samples were collected at RhizoKids International family conference for people affected by rhizomelic chondrodysplasia punctata^25^ (RhizoKids International, rhizokids.com). A total of 539 profiled plasma samples were included in this study, including 414 samples from existing studies and 125 previously unpublished samples (see Table 1). Across all 539 samples, there was a range of 376–684 named, z-scored compounds and a range of 0–261 unnamed (“unknown”), z-scored compounds included for each sample. Compounds rarely present in normal reference blood samples cannot be z-scored. However, argininosuccinate only presents in diseased samples and may serves as a strong predictor for disease and was thus encoded as a binary predictor (“1” for the presence or “0” for the absence). Unknown metabolites were excluded from the analysis in this work because the clinical utility of these compounds for diagnosis is limited.

**Table 1:** Description of data and data sources.

Disease (OMIM)	Disease gene	Related genes	Plasma profiles	Platform	Whole blood anti-coagulant	Used to learn network	References
Adenylosuccinase deficiency (MIM:103050)	*ADSL*		3	GC–MS, LC–MS^+,−^, MS^n^	EDTA	YES	Donti et al.^8^
Argininemia (MIM:207800)	*ARG1*		13	GC–MS, LC–MS^+,−,polar,lipid^, MS^n^	EDTA	YES	Burrage et al.^7^
			4	GC–MS, LC–MS^+,−,polar,lipid^, MS^n^	Heparin	NO	Miller et al.^5^
Argininosuccinic aciduria (MIM:207900)	*ASL*		11	GC–MS, LC–MS^+,−,polar,lipid^, MS^n^	EDTA	YES	Burrage et al.^7^
			2	GC–MS, LC–MS^+,−^, MS^n^	Heparin	NO	Miller et al.^5^
Aromatic l-amino acid decarboxylase deficiency (MIM:608643)	*DDC*		3	GC–MS, LC–MS^+,−^, MS^n^	EDTA	YES	Atwal et al.^9^, Pappan et al.^23^, Alaimo et al.^22^
Cerebral creatine deficiency syndrome 2 (MIM:612736)	*GAMT*		8	GC–MS, LC–MS^+,−^, MS^n^	Heparin	YES	Miller et al.^5^
Citrullinemia (MIM:215700)	*ASS1*	*SLC25A13*	9	GC–MS, LC–MS^+,−^, MS^n^	Heparin	YES	Miller et al.^5^, Burrage et al.^7^
Cobalamin biosynthesis defect (MIM:277400, 277410, 236270, 277380, 250940, 614857, 309541)		*MMACHC, MMADHC, MTRR, LMBRD1, MTR, ABCD4, HCFC1*	6	GC–MS, LC–MS^+,−^, MS^n^	Heparin	YES	Miller et al.^5^
GABA-transaminase deficiency (MIM:613163)	*ABAT*		7	GC–MS, LC–MS^+,−,polar,lipid^, MS^n^	EDTA	YES	Kennedy et al.^10^, Alaimo et al.^21^
Glutaric acidemia 1 (MIM:231670)	*GCDH*	*ETFA, ETFB, ETFDH, C7ORF10*	5	GC–MS, LC–MS^+,−^, MS^n^	Heparin	YES	Miller et al.^5^
Maple syrup urine disease (MIM:248600)		*BCKDHA, BCKDHB, DBT*	18	GC–MS, LC–MS^+,−^, MS^n^	Heparin	YES	Miller et al.^5^
Methylmalonic aciduria (MIM:251100, 251000)		*MMAA, MMAB, MUT, MMADHC, MCEE*	9	GC–MS, LC–MS^+,−^, MS^n^	Heparin	YES	Miller et al.^5^
Ornithine transcarbamylase deficiency (MIM:311250)	*OTC*		17	GC–MS, LC–MS^+,−^, MS^n^	EDTA	YES	Burrage et al.^7^
			17	GC–MS, LC–MS^+,−^, MS^n^	Heparin	NO	Miller et al.^5^
Phenylketonuria (MIM:261600)	*PAH*		8	GC–MS, LC–MS^+,−^, MS^n^	Heparin	YES	Miller et al.^5^
Propionic acidemia (MIM:606054)		*PCCA, PCCB*	9	GC–MS, LC–MS^+,−^, MS^n^	Heparin	YES	Miller et al.^5^
Rhizomelic chondrodysplasia punctata (MIM:215100)	*PEX7*	*GNPAT, AGPS, FAR1, PEX5*	21	GC–MS, LC–MS^+,−,polar,lipid^, MS^n^	EDTA	YES	This study
Zellweger spectrum disorder (MIM:214100, 601539)	*PEX1*	*PEX2, PEX3, PEX5, PEX6, PEX10, PEX11B, PEX12, PEX13, PEX14, PEX16, PEX19, PEX26, HSD17B4*	18	LC–MS^+,−,polar,lipid^, MS^n^	EDTA	YES	Wangler et al.^6^, Alaimo et al.^21^
Unknown	Unknown		185	GC–MS, LC–MS^+,−,polar,lipid^, MS^n^	EDTA	NO	Alaimo et al.^22^
Reference	*N/A*		104	GC–MS, LC–MS^+,−,polar,lipid^, MS^n^	EDTA	YES	This study
			68	GC–MS, LC–MS^+,−^, MS^n^	Heparin	YES	Miller et al.^5^

For each disease cohort, the number of samples and the publication source of the data are described. Individuals in the Unknown cohort^22^, a cohort whose clinical diagnoses from which we were blinded, may also have diagnoses associated with any of the disease cohorts listed. When samples in the Unknown cohort were identical to samples associated with a known clinical diagnosis, Alaimo et al.^22^ is also referenced. For samples from Miller et al.^5^, genetic sequencing data was not available and thus, only biochemical diagnoses were made. As a result, when more than one gene is responsible for a given diagnosis, all known genes associated with the given diagnosis are listed in the Related Genes column.

*GC–MS* gas chromatography–mass spectrometry, *LC–MS* liquid chromatography–mass spectrometry, *MS*^*n*^ multi-stage mass spectrometry.

### Inference of disease-specific networks

Metabolites with z-scores in > 50% reference samples and > 50% disease samples were used for network learning. Missing z-scores were imputed using the minimum z-score of the analyte in a large reference population. In order to model the differences in perturbation signatures between disease cases and controls, two types of Gaussian graphical network models were then learned from the data: one from both disease and control samples (disease + control network), and a second from only control samples (control network)^21^. We used the Graphical Lasso algorithm implemented in the R package huge (v1.3.5) to estimate the precision matrix, where regularization parameter lambda is selected using criteria “stars”. For both graphs, edge weights are the estimated partial correlation between any two metabolites after conditioning on all other variables in the datasets. Next, edges found in the disease + control network that were also found in the control network were pruned^21^. This pruned, “disease-specific” network represents probability of any pairs of metabolites being co-perturbed together at the state of the disease and was used in downstream analysis (Fig. 1). Including both examples of disease and control profiles in the training data (“discriminative latent structure inference”) introduces a hidden variable representing the disease state associated with each sample, allowing the network to model the specific metabolomic differences between two conditions (disease vs. control). Disease-specific network models from five IEMs in Thistlethwaite et al.^21^ were included, as well as novel network models learned on 11 additional IEMs, totaling 16 IEM disease states (Table S2). All network structures used in this paper are accessible through the CTDext R package accessible via GitHub (https://github.com/BRL-BCM/CTDext), an extension of the CTD CRAN R package that also includes added functionalities and filesharing necessary to reproduce our results. Graphical model metrics including node count, edge count and graph density for all network structures are documented in Table S2.

### The CTD method

**Figure 2:**
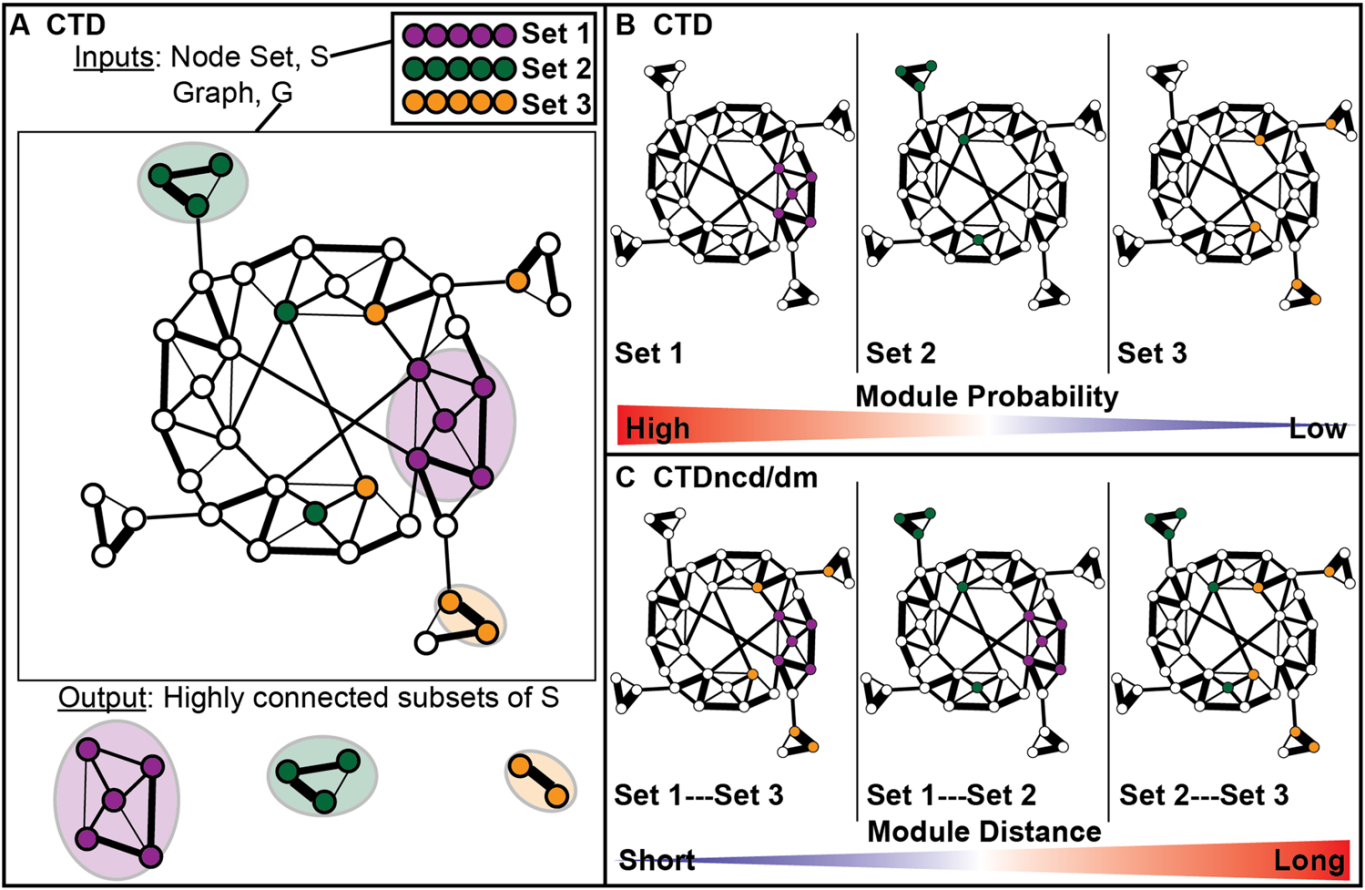
CTDncd and CTDdm extend CTD to quantify distances between two sets of nodes in a network. (**A**) CTD outputs highly connected subsets of a node set (given as an input) in a graph (also given as an input). (**B**) CTD assigns higher significance to highly connected node sets compared to sparsely connected node sets. (**C**) Node sets found in identical or neighboring regions in a graph are assigned shorter distances compared to node sets found in distal regions of a graph. CTDncd calculates distances between two individuals, where node sets being compared are based on observed metabolite perturbations in two individuals’ metabolomics profiles. CTDdm calculates distances between an individual and a disease state, where node sets being compared are the main disease module in a graph (see Algorithm 1 in Supplemental Text 1) and the observed metabolite perturbations in a single individual’s metabolomic profile.

The CTD method was described previously^21^. Briefly, the method takes a weighted, disease-specific “co-perturbation” network and a set of network nodes as inputs and identifies a subset of the input nodes that is highly connected within the network (Fig. 2A,B). The method also provides a p value corresponding to the level of connectedness of the input node subset within the network.

### The CTDncd distance: a network-based patient-patient distance method

In the context of a network, two individuals’ sets of metabolite perturbations can be compared by calculating the distance between them. If two metabolite sets hit the same nodes or modules in the context of a network, they may be considered related; however, if two metabolite sets share no overlapping nodes, nor do they hit proximal parts of a network, they may be considered unrelated (Fig. 2C). To calculate the distance between two node subsets in a network, we use the Normalized Compression Distance (NCD) metric^26^ which is based on normalized mutual information (Eq. 1).

1 $$CTDncd({S}_{1}, {S}_{2}) = \frac{max(I({S}_{1}, {S}_{2})-I({S}_{1}), I({S}_{1}, {S}_{2})-I({S}_{2}))}{max(I({S}_{1}), I({S}_{2}))}$$

### The CTDdm distance: a network-based patient-disease distance method, a variation on CTDncd

CTDdm also uses Eq. (1) to calculate the distance between a set of metabolites perturbed in an individual and the metabolite set perturbed in individuals with a specific disease (Fig. 1). This set, referred to as the “main disease module”, is calculated by Algorithm 1 in Supplemental Text 1. Information provided by CTDdm was shown to reduce false positive rate and improve overall diagnostic accuracy when combined with the CTD method (Table S1). CTD + CTDdm metrics were therefore used for scoring sample profiles.

### Analysis of exome sequencing data

The collection and processing of clinical exome sequencing data from 170 individuals is detailed in Alaimo et al.^22^. Briefly, data were acquired using protocols adapted for clinical testing, described previously^27^. Variants were called using AtlasSNP2^28^ (v. 1.4.3). Variants in intronic or intergenic regions were filtered out, as well as variants found in ESP5400 or 1000 Genomes^29^ at frequencies greater than 0.05. The pathogenicity of each genetic variant was assessed according to the ACMG/AMP guidelines^3,30^. For each disease gene associated with any of the 15 IEMs (X-linked OTC deficiency excluded) modeled in this paper, we assigned each individual to one of 5 classes (Table 2) based on the assumption of an autosomal recessive inheritance pattern, the ACMG/AMP pathogenicity category, and observed zygosity of the variants in individuals’ exomes.

**Table 2:** Categorization of individuals based on classification of genetic variants identified in personal genome data.

Class	Interpretation	Variants identified
1	Disease case	At least 2 known heterozygous pathogenic or 1 homozygous pathogenic
2	At least a carrier	1 known heterozygous pathogenic and at least 1 heterozygous VUS
3	Uncertain	At least 1 homozygous VUS or at least 2 heterozygous VUSs
4	Potential carrier	Exactly 1 heterozygous VUS
5	Control	All benign

For each gene known to cause a given IEM, variants identified in a personal genome were assigned a pathogenicity category based on the application of the ACMG/AMP guidelines. Secondly, the observed zygosity (e.g., heterozygous, hemizygous or homozygous) of the variants identified in an individual’s exome was considered alongside the expected Mendelian mode of inheritance for the disease gene (i.e., autosomal recessive).

### The metabolomics data portal: a diagnostics tool for untargeted metabolomics data

To provide access to the data and to provide a prototype tool to aid in the clinical diagnostic process, we developed an R shiny application. This application visualizes individuals’ metabolomics data and implements the network-assisted diagnostic functionalities featured in this paper. A walkthrough of the features this application offers can be found in Supplemental Text 1. The full application can be accessed at https://genboree.org/genboreeKB/projects/metabolomics-data-portal.

## Results

### Data-driven network models show higher accuracy relative to rule-based biomarker models and eliminate the requirement for a priori biomarkers

Prior knowledge, including lists of metabolite biomarkers, have previously been integrated into rule-based models for diagnosis in metabolomics^20^. Network-based modeling is less biased toward specific lists of biomarkers and models the co-variation between metabolites and is thus more suitable in the discovery and complex diagnostics contexts. We explored the possibility that network-based approaches may also show added accuracy associated with incorporating information from full untargeted metabolomics profiles compared to information based solely from known biomarkers of metabolic disease states.

Haijes et al.^20^ reported models that rank diseases for each individual based on lists of known biomarkers for each IEM. The correct diagnosis ranked first in 37% of 115 validation plasma samples. In 72% of cases, the correct diagnosis could be found within a short “differential diagnosis” (DD) list of candidate diagnoses, returned by the rule-based algorithm. To compare this rule-based method to CTD, we assigned a DD to an individual based on whether combined network-quantified scores (CTD + CTDdm) match at a Bonferroni-corrected combined network p value < 0.05 in the corresponding disease-specific network model.

As shown in Table 3, CTD + CTDdm ranked the correct diagnosis (from 16 modeled IEMs) first in 70% of 154 samples. Moreover, 89% of samples had the correct diagnosis in their DD short list. When we omitted individuals with OTC deficiency (MIM:311250)—where 13 out of 17 were female—from consideration due to diagnostic difficulties associated with the possibility of skewed X-inactivation in females with this diagnosis^31,32^, CTD + CTDdm ranked the correct diagnosis first in 79% of the remaining 137 samples with known diagnoses across the remaining 15 modeled IEMs (Table 3), and 94% of samples had the correct diagnosis in their DD short list. Prediction performance (sensitivity, specificity, accuracy) of all individual disease-specific models measured by CTD + CTDdm ranks is shown in Table S3. All disease rankings for each individual can be viewed using the Metabolomics Data Portal (see “Methods”), in the Network-assisted Diagnostics tab (Figure S2b).

**Table 3:** Accuracy of diagnostic rankings across 188 plasma samples with known disease.

Diagnostic method	# IEM	Length DD (median, 5th-, 95th-percentile)	% Top 1	% Top 3	% in DD
Haijes et al.^20^	58	10 [3–22] (out of 58)	0.37	0.57	0.72
CTD + CTDdm	16	3 [1–8] (out of 16)	0.70	0.87	0.89
CTD + CTDdm	15	3 [1–7] (out of 15)	0.79	0.94	0.94

A differential diagnosis list (DD) is a ranked list of potential “candidate” diagnoses for each individual. Diagnoses were added to the DD if individual sample data meet a given threshold defined by each diagnostic method. Rankings were determined and compared for both a rule-based method described in Haijes et al.^20^ and for our combined network (CTD + CTDdm) approach.

*IEMs* inborn errors of metabolism, *DD* differential diagnosis list.

To determine whether the increased sensitivity comes at the cost of lower specificity, we also examined the median size of the DD short lists for 172 reference samples. Given that reference samples represent individuals without disease, a maximally specific method would assign 0 DDs to each of these individuals. In Haijes et al.^20^, reference samples identified a median of 3 out of 58 DDs. In our network-based approach, we found the median size to be 1 out of 16 DDs, verifying that our approach shows comparable specificity.

### Network approaches may be used for diagnosis in place of pathway-based approaches

While multi-metabolite perturbations are often interpreted in the context of well-curated pathway knowledge, such knowledge is not always available, does not include all measurable metabolites, and is not disease-specific. We therefore asked if data-driven network models may be used *in lieu* of pathway-based models. The possibility of using data-driven networks is particularly relevant for metabolic diseases whose affected pathways are not fully characterized, such as various peroxisomal, mitochondrial, and seizure disorders.

Toward this purpose, we defined a pathway-based diagnostic model to be one that considers only metabolites that are a priori known to be involved in a disease-relevant pathway, without information provided by the remaining untargeted metabolomics profile. In contrast, network-based diagnostic models and full-profile models were defined to be ones that considers all frequently detected metabolites in untargeted metabolomics profiles, without a priori information about which metabolites are most relevant for diagnosis. The former utilizes CTD-based metrics, whereas the latter includes all frequently detected metabolites as predictors.

**Figure 3:**
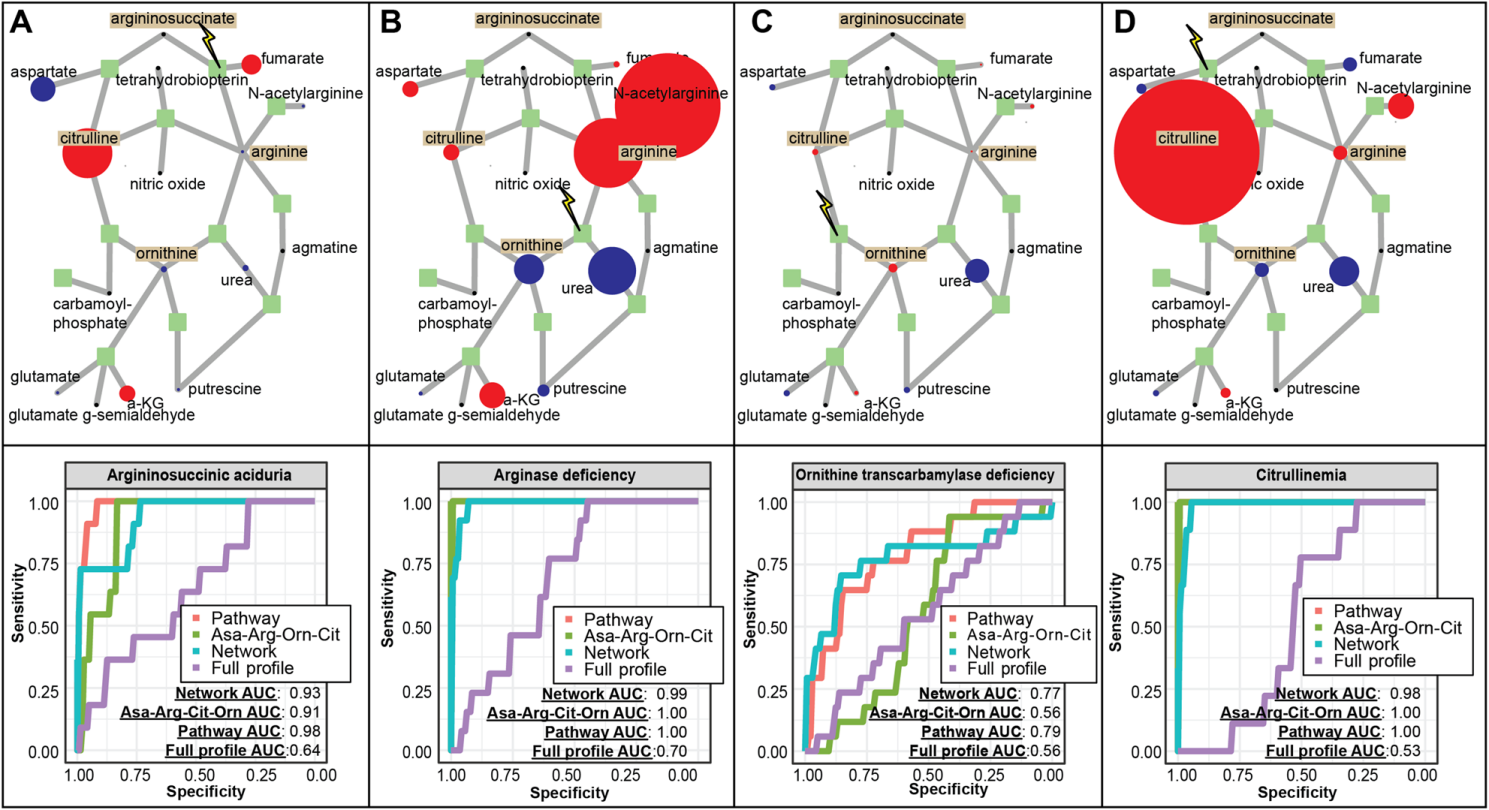
Data-derived networks are competitive with metabolic pathways as background knowledge network representations. For all models, (**A**) argininosuccinic aciduria, (**B**) argininemia, (**C**) ornithine transcarbamylase deficiency and (**D**) citrullinemia, the mean profile for each urea cycle disorder cohort is overlaid onto the urea cycle pathway. Red denotes a positive and blue denotes a negative perturbation, where the radius of the circle is modulated to reflect the magnitude of the perturbation. Below each urea cycle pathway, receiver-operator curves (ROC) between the two pathway-based models “ASA-Arg-Orn-Cit”, “Pathway” and the full-profile model are compared to the “Network” model.

To compare the approaches directly, we used untargeted metabolomics profiling data from individuals diagnosed with any of four genetically distinct urea cycle disorders^7^, where the disease mechanism is well-characterized by defects in enzymes and perturbations of metabolites in the urea cycle pathway (Fig. 3), as well as negative control (“reference”) profiles. We compared the performance of partial least squares regression models: two modeled the relative abundances of metabolites in the urea cycle; third modeled CTD- and CTDdm-quantified scores; fourth modeled all frequently detected metabolites in the untargeted metabolomics profiles. We note that argininosuccinate is rarely identified by our platforms due to its low concentration in normal plasma samples. As a consequence of its rare presentation in normal “reference” blood samples, z-scores for argininosuccinate levels cannot be generated when identified in an individual sample and thus, cannot be used as a quantitative predictor. Instead, we encoded argininosuccinate as a binary predictor (“1” for the presence or “0” for the absence of the metabolite). We, therefore, defined the “ASA-Arg-Orn-Cit” model to include quantitative variables, arginine, ornithine, and citrulline, and one binary variable, argininosuccinate. To further evaluate diagnostic accuracy, we defined the full “Pathway” model to include all metabolites found in both the urea cycle and the periphery of the urea cycle, as illustrated in Fig. 3.

As shown in Fig. 3, both network- and pathway-based models improve diagnostic power relative to the full-profile model. CTD performed competitively with pathway-based models of four major urea cycle disorders: argininosuccinic aciduria (MIM:207900), argininemia (MIM:207800), ornithine transcarbamylase (OTC) deficiency (MIM:311250), and citrullinemia (MIM:215700). In the case of argininosuccinic aciduria and particularly for OTC deficiency, the network-based model outperformed the ASA-Arg-Cit-Orn model and was competitive with the full Pathway model. Interestingly, model accuracies for OTC deficiency suffered from poorer discrimination compared to argininosuccinic aciduria, argininemia, and citrullinemia. This is partially due to the known phenotypic heterogeneity of effects associated with X-inactivation patterns observed in females with OTC deficiency^31,32^.

Overall, this result suggests network-based and pathway-based modeling approaches have comparable accuracies. Thus, when biochemical pathway knowledge for a particular disease state is not available, data-driven network-based models may provide a valuable alternative.

### Separating treatment-related effects from disease-related effects in metabolomics data

**Figure 4:**
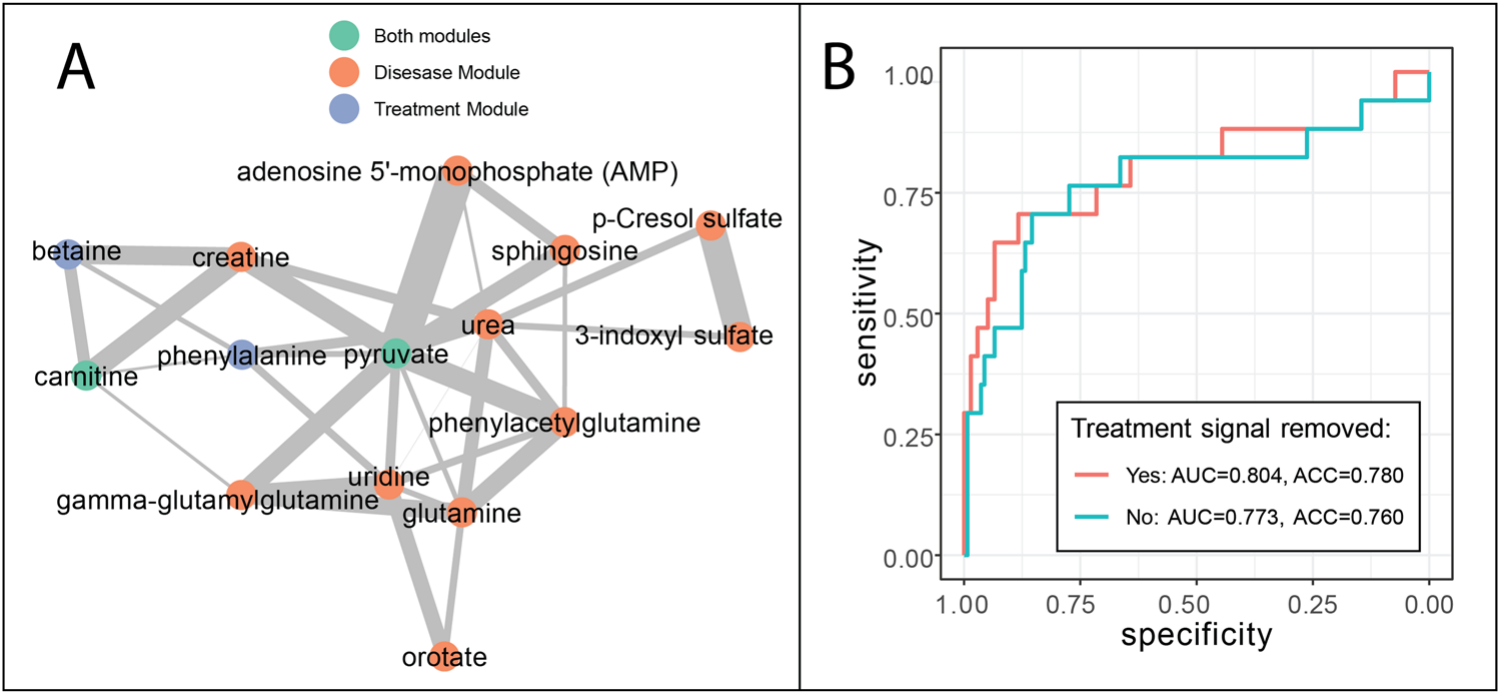
Impact of citrulline supplement on disease-specific network modeling ornithine transcarbamylase (OTC) deficiency. (**A**) Treatment module overlaps with main disease module for OTC deficiency and are well-connected. (**B**) Prediction performance of OTC deficiency model before and after removal of treatment module.

While the heterogeneity of effects associated with X-inactivation in OTC deficiency can explain why diagnostic accuracy of OTC deficiency was lower than many other IEMs, it is also likely that treatment-related effects may be confounding the raw metabolomics data and as a result, be affecting the ability of both pathway (knowledge-driven) and network-based (data-driven) models from performing well in diagnosis. To test for treatment confounding and whether it could be removed or ameliorated, we examined all OTC deficiency samples from Burrage et al.^7^ where treatment information is available. We found that 8/10 patients with OTC deficiency were taking citrulline supplements as part of a prescribed treatment regimen. To identify treatment-driven signatures that differentiated from disease-driven signatures, we constructed a citrulline supplement-specific network by contrasting eight OTC deficiency patients taking supplemental citrulline against the remaining 20 urea cycle disorder patients. A second network was learned from OTC deficiency patients not undergoing citrulline supplementation and edges found in both networks were pruned from the first network. Similar to “main disease module” identification (Algorithm 1), we determined the representative “main treatment module” for citrulline supplementation. When we compared the main disease module identified in the OTC disease-specific network to the main treatment module identified in the citrulline supplement-specific network, we found two out of four treatment-related compounds (e.g., carnitine and pyruvate) in the original OTC deficiency main disease module (Fig. 4A).

We then asked if treatment-related signatures contributed to false positives in patients with these diseases. Notably, the citrulline supplement-related treatment module was also found to overlap perturbations in other disease states such as cobalamin biosynthesis defect (carnitine, betaine) and argininosuccinate lyase deficiency (phenylalanine and pyruvate). As a consequence, we expected diagnostic accuracy to improve by omitting these treatment-related metabolites from the disease-specific OTC network folds. One case, for instance, is argininosuccinate lyase deficiency patient “EDTA-ASLD-7”, where OTC deficiency was falsely ranked first by CTD + CTDdm (combined, p = 0.00568), while the correct diagnosis fell out of the top three rankings (combined, p = 0.0385). With the updated network model, significance of OTC deficiency dropped to the 5th in the list of diagnoses (combined, p = 0.0716), and the correct diagnosis ascended to within top three. Additionally, removal of treatment signal also unflagged a cobalamin biosynthesis defect patient “EDTA-COB-6”, as the significance of OTC deficiency dropped an order of magnitude (combined, p = 0.00335). Removal of treatment-related compounds also increased model AUC, specificity, and overall accuracy (Fig. 4B).

This result suggests that pruning treatment-related nodes can improve the diagnosis of patients with other diseases where compounds to be removed are affected by the disease state. Nevertheless, the pruning of treatment-related nodes should be performed with caution, as treatment-related signatures that are particularly specific to a given diagnostic category can also improve diagnostic performance. Ideally, we would only collect metabolomic data prior to administering treatment, in order to train disease-specific network models for diagnostic purposes. However, many individuals that undergo untargeted metabolomic screening are already on a treatment regimen in order to manage their symptoms, and as a result, the confounding due to treatment is common. While we have shown that censoring treatment-related metabolites from our diagnostic OTC network models helped diagnostic accuracy, this represents only a first pass attempt to separate treatment effects from disease effects. Future research may be needed to explore new strategies that would improve diagnostic accuracy by removing treatment effects.

### Dissecting the genetic etiology of peroxisome biogenesis disorders using untargeted metabolomics with network models

Peroxisome biogenesis disorders (PBDs) are autosomal recessive disorders that result from the impaired assembly and biological functioning of peroxisomes^33^. They are composed of two known major classes: Zellweger spectrum disorders (ZSD, MIM:214100,601539) and rhizomelic chondrodysplasia punctata (RCDP, MIM:215100). As a whole, PBDs are complex since they are caused by deficiency of any single gene in a group of related genes and their severity is strongly influenced by the residual activity of downstream gene products. Furthermore, PBDs affect the functions of numerous organ systems, and their pathological mechanisms of disease are only partially understood. We therefore asked whether a data-driven network-based approach may help dissect their etiology and help provide diagnostic information.

**Figure 5:**
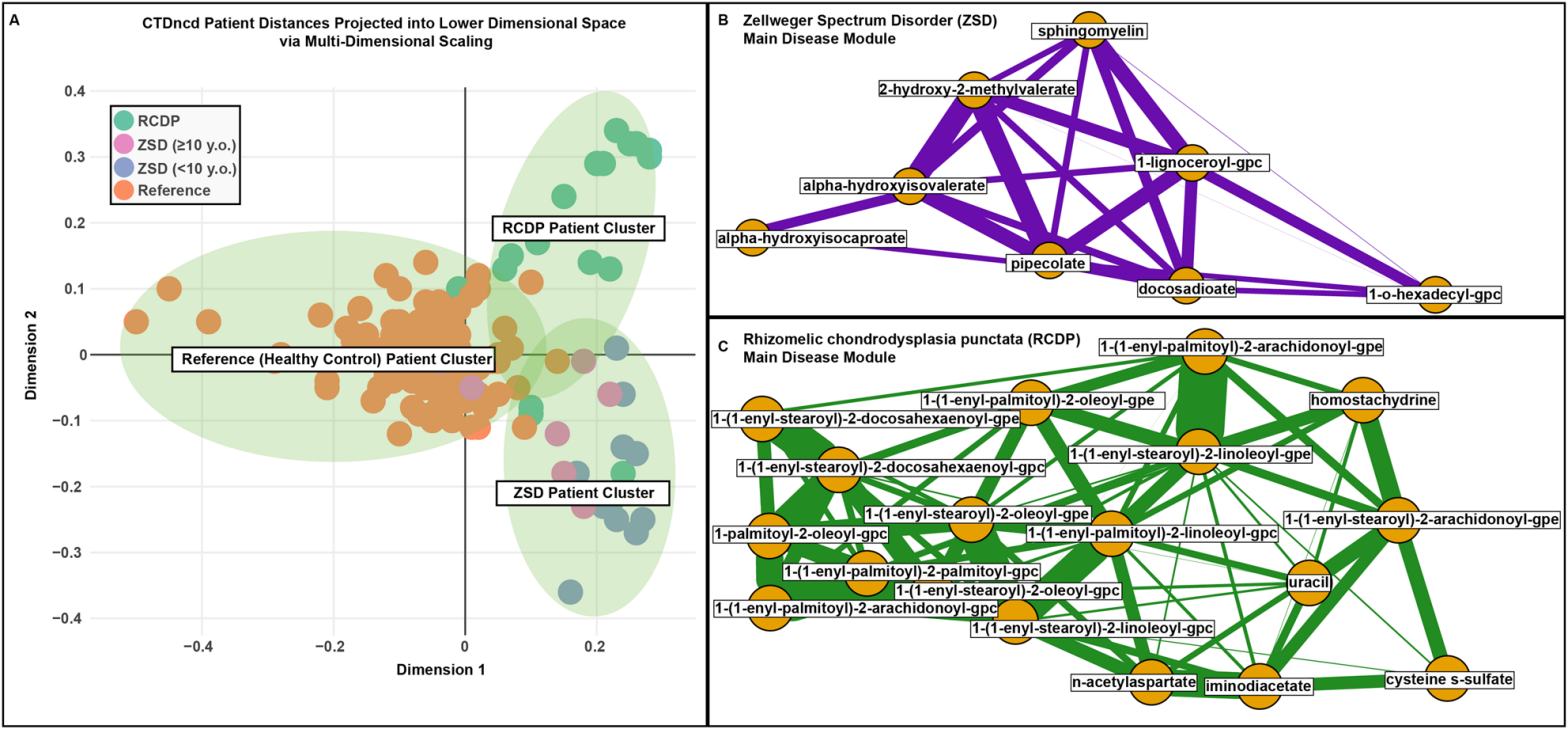
Zellweger spectrum disorder (ZSD), rhizomelic chondrodysplasia punctata (RCDP) and reference (REF) individuals cluster by disease using CTDncd. (**A**) Dots represent individual samples in a lower dimensional 2-D space using multi-dimensional scaling. Individuals are colored by their diagnostic state (e.g., ZSD samples in pink and blue, RCDP samples in green, and reference samples in orange). Within the ZSD cluster, age-related effects can be identified whereby the older individuals with the disease ($\ge$ 10 years old) generally show less pronounced abnormalities in metabolite levels, in agreement with Wangler et al.^6^, while younger patients (< 10 years of age) showed greater heterogeneity in this regard. (**B**,**C**) Main disease modules for ZSD and RCDP, respectively.

Untargeted metabolomic profiling from Wangler et al.^6^ describe 18 individuals with ZSD originating from deleterious variants in *PEX1* (MIM:602136). We collected an additional 21 samples from individuals diagnosed with a RCDP type 1 disorder with confirmed deleterious variants in *PEX7* (MIM:601757). For all 39 samples, pairwise patient-patient distance calculations using CTDncd were estimated and plotted in lower dimensional space (Fig. 5). As shown, the distances accurately cluster individuals with ZSD separately from individuals with RCDP and from known reference samples. Interestingly, examination of the ZSD cluster highlighted less pronounced abnormalities in plasma metabolite levels in older individuals ($\ge$ 10 years) with a smaller centroid–centroid distance to the reference cluster (d = 0.625) than that of younger patients (< 10 years of age) (d = 0.949), in agreement with Wangler et al.^6^. Overall, k-means clustering generated a cluster purity score of 0.888, which indicates low false positive and false negative clustering results. These results suggest that CTDncd can accurately distinguish between groups of individuals with ZSD and RCDP, the two major subtypes of PBDs.

### Evidence from the metabolome can help interpret variants of uncertain significance

Methods for using untargeted metabolomics data to resolve the pathogenicity of VUSs are currently manual, qualitative, and can be very laborious. We therefore asked if disease-specific networks and CTD-based metrics may help automate the interpretation of untargeted metabolomics profiling data to improve variant assessment. We recently reported that manual evaluation of untargeted metabolomics data aided in the diagnosis in the context of exome sequencing for several IEMs^22^. In our current analysis, we interpreted the same exome sequencing and respective metabolomics data in two stages. First, as in Alaimo et al.^22^, genetic variants identified in patient exome data were classified according to the ACMG/AMP guidelines. Individuals with variants found in at least one disease gene relevant to any of the 15 IEMs (X-linked OTC excluded) modeled in our analysis were categorized into the one of 5 groups (Table 2) based on the observed variants’ pathogenicity and zygosity. Second, select patient metabolomics data were then analyzed using CTD and CTDdm in the context of relevant IEM-specific networks.

For all individuals with variants categorized into groups 1–3 (Table 2) in disease-relevant genes for a given disease state (Table 1), individuals with a combined CTD + CTDdm disease-specific network p value < 0.05 were reported as a HIT, and the pathogenicity of the respective variants was re-interpreted towards a more pathogenic classification. Furthermore, individuals with combined network p values between 0.05 and 0.15 were reported as a BORDERLINE HIT. Of all 29 variant confirmations or re-interpretations reported by Alaimo et al.^22^, 10 had variants in disease genes relevant to the 15 IEMs (X-linked OTC excluded) modeled in this paper. Of all 10 individuals, 9/10 were classified as HIT and 1/10 was a BORDERLINE HIT (Table 4). These results suggest that the interpretation of untargeted metabolomic profiling data to improve variant assessment can be automated using CTD-based metrics.

**Table 4:** Variant re-interpretations based on evidence quantified from the metabolome.

Pt	Sex	Age	Variant	AA	Zyg	OMIM	Net. (Rank)	CTD	CTDdm	Comb	Module detected (Z-score)	ACMG
**10**	F	21	*PEX16*, CA250385, NM_004813.4: c.993_995del	N/A	Hom	614862; 614863	Zellweger spectrum disorder (6/16)	0.002	0.63	0.009	16-Hydroxypalmitate (2.306)	LP to P
											3-Hydroxylaurate (2.494)	
											5-Dodecenoate (12:1n7) (2.017)	
											Alpha-hydroxyisovalerate (1.908)	
											Docosadioate (1.843)	
											Nonadecanoate (19:0) (1.843)	
**44**	M	2	*DDC*, CA367529579, NM_000790.4: c.286G > A	G96R	Het	608643	Aromatic l-amino acid decarboxylase deficiency (1/16)	2e−16	0.07	5e−16	3-Methoxytyrosine (6.081)	VUS to LP
											9,10-Dihome (2.202)	
											Adipate (− 3.598)	
											Deoxycholate (− 2.510)	
											Gamma-Glutamyltyrosine (− 2.942)	
											Hydroquinone sulfate (2.716)	
											Indoleacetate (2.315)	
											Kynurenate (− 2.517)	
											Pipecolate (1.716)	
			*DDC*, CA4262432, NM_000790.4: c.260C > T	P87L	Het						Pyroglutamylleucine (2.659)	VUS to LP
											s-Methylcysteine (3.599)	
											Taurochenodeoxycholate (− 1.810)	
											Taurocholate (− 2.305)	
											Taurodeoxycholate (− 1.981)	
											Taurolithocholate 3-sulfate (− 2.198)	
											Tryptophan betaine (− 2.279)	
											Vanillylmandelate (vma) (− 2.708)	
**48**	F	1	*PAH*, CA229811, NM_000277.3: c.842 + 1G > A	N/A	Het	261600	Phenylketonuria (1/16)	4e−7	0.02	1e−7	Arachidonate (20:4n6) (− 1.626)	Confirms (P)
											Docosahexaenoate (dha; 22:6n3) (− 1.769)	
											erucate (22:1n9) (− 2.209)	
			*PAH*, CA229775, NM_000277.3: c.805A > C	I269L	Het						Gamma-glutamylphenylalanine (2.328)	VUS to LP
											Myristoleate (14:1n5) (− 1.665)	
											n-Acetylphenylalanine (1.801)	
											Palmitate (16:0) (− 2.024)	
											Palmitoleate (16:1n7) (− 1.696)	
											Phenylalanine (3.452)	
											Stearate (18:0) (− 2.247)	
**55**	M	15	*GAMT*, CA295620, NM_000156.6: c.79 T > C	Y27H	Hom	612736	Cerebral creatine deficiency syndrome 2 (14/16)	2e−2	0.54	6e−2	2-Hydroxyglutarate (2.500)	VUS to LP
											Creatine (− 3.048)	
											Pyroglutamine (2.314)	
**68a**	M	1	*ABAT*, CA394688322, NM_020686.6: c.454C > T	P152S	Het	613163	GABA-transaminase deficiency (3/16, 1/16)	7e−5	0.04	4e−5	2-Pyrrolidinone (6.883)	VUS to LP
											4-Guanidinobutanoate (2.110)	
											4-Methyl-2-oxopentanoate (2.410)	
											Isoleucine (1.490)	
											Leucine (1.997)	
											Lysine (1.552)	
**68b**			*ABAT*, CA394691458, NM_020686.6: c.1393G > C	G465R	Het			5e−6	0.05	4e−6	2-Pyrrolidinone (6.157)	VUS to LP
											4-Guanidinobutanoate (2.514)	
											Caprylate (8:0) (3.767)	
											Creatinine (− 1.984)	
											Glucuronide of c10h18o2 (2.529)	
											Maleate (cis-butenedioate) (3.475)	
											n-Acetylmethionine (6.650)	
											Tauroursodeoxycholate (3.475)	
**85**	M	4	*ABAT*, CA394692408, NM_020686.6: c.168 + 1G > A	N/A	Het	613163	GABA-transaminase deficiency (1/16)	3e−7	0.01	3e−8	1-Linoleoylglycerol (1-monolinolein) (1.954)	Confirms (P)
											2-Pyrrolidinone (2.196)	
											4-Guanidinobutanoate (3.028)	
											Cis-4-decenoyl carnitine (− 1.845)	
			*ABAT*, CA394688780, NM_020686.6: c.638 T > G	F213C	Het						Decanoylcarnitine (− 2.552)	VUS to LP
											Iminodiacetate (ida) (− 2.599)	
											Myristoylcarnitine (− 2.752)	
											Sphinganine (2.001)	
											Sphingosine (2.561)	
**92**	M	1	*PEX6*, CA3811598, NM_000287.4: c.611C > G	S204*	Hom	614862; 614863	Zellweger spectrum disorder (2/16)	8.3e−16	0.02	7.9e−16	1-(1-Enyl-palmitoyl)-2-linoleoyl-gpe (p-16:0/18:2) (− 4.237)	Confirms (P)
											1-(1-Enyl-palmitoyl)-2-oleoyl-gpc (p-16:0/18:1) (− 3.976)	
											1-(1-Enyl-palmitoyl)-2-palmitoleoyl-gpc (p-16:0/16:1) (− 3.700)	
											1-(1-Enyl-palmitoyl)-2-palmitoyl-gpc (p-16:0/16:0) (− 3.912)	
											1-(1-Enyl-stearoyl)-2-arachidonoyl-gpe (p-18:0/20:4) (− 4.272)	
											1-(1-Enyl-stearoyl)-2-docosahexaenoyl-gpc (p-18:0/22:6) (− 4.349)	
											1-(1-Enyl-stearoyl)-2-linoleoyl-gpe (p-18:0/18:2) (− 6.191)	
											1-Lignoceroyl-gpc (24:0) (6.100)	
											1-o-Hexadecyl-gpc (c16) (− 5.368)	
											1-Oleoyl-2-docosahexaenoyl-gpc (18:1/22:6) (− 4.415)	
											1-Palmitoleoyl-2-linoleoyl-gpc (16:1/18:2) (− 5.012)	
											1-Palmityl-2-oleoyl-gpc (o-16:0/18:1) (− 7.014)	
											2-Hydroxy-3-methylvalerate (6.160)	
											Alpha-hydroxyisovalerate (4.571)	
											Docosadioate (4.096)	
											Hexadecanedioate (5.136)	
											Phenyllactate (pla) (4.164)	
											Pipecolate (5.901)	
											Sphingomyelin (− 4.565)	
											Sphingomyelin (d18:1/17:0, d17:1/18:0, d19:1/16:0) (− 4.108)	
**136**	F	< 1	*DDC*, CA4262295 NM_001082971.2: c.714 + 4A > T	N/A	Hom	608643	Aromatic l-amino acid decarboxylase deficiency (1/16)	5.3e−03	0.04	1.8e−03	3-Methoxytyrosine (6.059)	Confirms (P)
											Cortisol (− 4.380)	
											Cortisone (− 3.736)	
											Gamma-glutamyltyrosine (− 3.164)	
											Glucose (6.795)	
											Glucuronate (− 4.052)	
											Indoleacetate (− 5.349)	
											o-Sulfo-l-tyrosine (− 4.879)	
											Succinate (− 5.165)	
											Vanillylmandelate (vma) (− 3.367)	
**146**	M	< 1	*MTR*, CA345379301 NM_000254.3: c.2405 + 1G > A	N/A	Het	250940	Cobalamin biosynthesis defect (4/16)	1.6e−04	0.02	4.0e−05	2-Aminooctanoate (− 3.202) 3-Indoxyl sulfate (− 7.687) betaine (10.549) Dimethylglycine (4.747) n-Acetylphenylalanine (3.049) Phenylacetylglutamine (− 2.573)	Confirms (P)
			*MTR*, CA923726079 NM_000254.3: c.2473 + 3A > G	N/A	Het							LP to P
**157**	F	1	*ASS1*, CA375229529 NM_000050.4: c.830A > G	K277R	Hom	215700	Citrullinemia (5/16)	7.0e−03	0.15	8.5e−03	Arachidonate 20:4n6 (− 1.633)	LP to P
											Citrulline (+ 7.086)	
**166**	M	< 1	*MUT*, CA138796356 NM_000255.4: c.1218delG	N407fs	Het	251000	Methylmalonic aciduria (4/16)	4.3e−03	0.22	7.4e−03	1-Pentadecanoylglycerophosphocholine 15:0 (+ 2.353)	Confirms (P)
			*MUT*, CA3846855NM_000255.4: c.1531C > T	R511*	Het						1-Margaroylglycerophosphoethanolamine (+ 2.902)	Confirms (P)

10/10 variant interpretations discovered by manually inspecting metabolomics data from 170 individuals in Alaimo et al.^22^ were reproduced using our automated pipeline, where 9/10 of those had strong significance and 1/10 had borderline significance. One novel finding is also reported, where one individual was diagnosed with a PBD, highlighting the ability of CTD-based metrics to detect disease-relevant signatures that are too complex or subtle to detect using manual inspection.

*Pt* patient, *AA* amino acid change, *Zyg *zygosity, *OMIM* diagnosis identifier from the Online Mendelian Inheritance in Man catalog, *Net* disease-specific network, *Comb* Brown’s combined p value, *ACMG* The American College of Medical Genetics variant pathogenicity classification.

Of further relevance are the patterns detected by CTD + CTDdm that connect individuals to disease states and that escaped manual inspection in Alaimo et al.^22^ (Table 4). Patient 10 was a 21-year-old female who had a likely pathogenic homozygous variant (NM_004813.4:c.993_995del) in *PEX16* (MIM:603360). Her metabolomic profile showed several metabolite perturbations consistent with a PBD. Out of 16 diagnoses, ZSD ranked 6th and RCDP ranked 2nd. CTD detected a module containing a very long-chain fatty acid that was positively perturbed (Table 4), a hallmark of ZSD, in the ZSD disease network. Review of clinical reports revealed that Patient 10 had an intellectual disability, spasticity, ataxia and structural brain abnormalities, phenotypes consistent with those observed in individuals with *PEX16* pathogenic variants, as reported previously^34^. We then confirmed that this individual was the same individual who was diagnosed with a PBD in a recent publication^35^, a diagnosis that took over 18 years to establish. We previously reported that plasma disease signatures in individuals with a mild to intermediate ZSD are more pronounced in younger subjects, suggesting studies earlier in life reveal larger biochemical changes for a number of possible reasons^6^. CTD’s ability to detect ZSD-relevant disease signatures in Patient 10 (21-year-old), however, shows how CTD can assist clinicians in difficult diagnostic situations.

While the CTDdm score put Patient 10’s module moderately far away from the main disease module (63rd percentile), the ZSD disease network modeled perturbation patterns in individuals with *PEX1* defects from a variety of different levels of severity. It is therefore plausible that a mild *PEX16* disease signature, while highly connected in the *PEX1* network, was more distal to several metabolites perturbed in individuals with *PEX1*-associated ZSD. To make the ZSD disease network more sensitive to disease signatures observed in each of the 14 *PEX* genes that cause ZSD, using profiling data from individuals with biallelic defects in any of several different *PEX* genes would be beneficial for network learning. While this diagnosis was further complicated by reduced plasma-derived perturbations with age observed in ZSDs, the fact that the diagnosis was missed by previous manual inspection of metabolomic data^22^, however, highlights the power of our data-driven network method to identify both the modeled (*PEX1*) and related disease gene’s (*PEX16*) effect on the metabolome.

## Discussion

Several recent publications have called for systems biology solutions to shortcomings observed in current diagnostic methods for metabolic disorders^36–38^. IEMs provide a useful context for testing novel computational approaches such as CTD because the genetic etiology of many IEMs is well-established. In this work, we have shown the accuracy of the CTD method using untargeted metabolomics on a variety of IEM data sets. Our results pave the way toward the dissection of the genetic etiology and precise diagnosis of more complex, metabolically heterogeneous diseases, such as diabetes and metabolic syndrome.

CTD characterizes individuals’ metabolomic likeness to a given disease state based on the connectedness of metabolite perturbations in a disease-specific network. Analogous to the background knowledge clinicians accumulate about metabolism by combining textbook knowledge of biochemistry and experience in the clinic, disease-specific networks are learned directly from representative profiling data and reflect information found in well-curated pathway knowledge^39^. Network-based similarity metrics have been constructed previously for use in disease-disease similarity^40^, functional protein similarity^41^, and similarity in clinical ontological terms^42^. We apply similar logic to quantify patient-patient (CTDncd) or patient-disease (CTDdm) distances using metabolomics data and use both metrics as additional network-quantified predictors of diagnosis.

CTD makes few assumptions about the nature of disease-associated perturbations and is thus well-suited for discovery and diagnosis of hard-to-diagnose cases. For example, CTD does not make any hard assumptions about the metabolites involved in a particular disease or the directionality (+ or −) of perturbations. While information about the directionality of a metabolite perturbation can be useful for diagnostic discrimination, there are several situations where this information can be disadvantageous. For example, related disease states and disease genes frequently affect the same molecular components but in different ways^40,43^. Just as different variants identified in a single gene can lead to different levels of disease severity, modeling the effect that one disease gene has on a metabolic phenotype may not accurately predict the metabolic effect of another gene that is involved in the same pathway, particularly when it comes to the direction that specific metabolites are perturbed. By considering combinatorial patterns without regard to directionality, CTD-based metrics make fewer assumptions and are therefore more suitable for data-driven discovery and for resolving hard-to-diagnose cases. If routine discrimination and not discovery is the main goal, information about the directionality of metabolite perturbations can be combined with combinatorial information garnered in CTD-based metrics (see Supplemental Text 1).

As is the case with any data-driven modeling approach, the performance of CTD-based metrics is mostly determined by the amount and quality of data available. Based on our experience, we recommend a minimum of 5 disease profiles, a minimum of 25 reference profiles for each disease condition, and the use of surrogate disease and negative control profiles^21^ to learn stable disease-specific networks (Figure S5). If the samples represent a spectrum of disease severity or are the result of defects from more than one gene, we recommend gathering even more unique examples of disease prior to network learning.

The paucity of profiles available for rare disorders similarly affects both our network-based and rule-based approaches. There are many metabolic disease states including several IEMs where the paucity of cases has precluded data-driven classification. When a new case of such diseases shows a different metabolic perturbation signature that is contrary to existing biomarker-based knowledge, the case is very likely to be falsely omitted from the correct diagnosis by rule-based algorithms^20^. This problem will only be alleviated with the accumulation of more case profiling data. One strength of the network-based strategy is that new data can be incorporated automatically by a structured network learning process. In contrast, for rule-based approaches, knowledge curation and optimization can often be laborious.

Network models may also be significantly affected by confounding factors. For example, networks learned from samples collected after treatment was initiated will inevitably connect both treatment-related and disease-related metabolites together. Treatment-related modules, if not pruned properly in the network pruning stage, can cause some individuals without the modeled disease to be falsely diagnosed. CTDdm is designed to identify false positive calls made by CTD alone (see Supplemental Text 1) but fails when the treatment-related module is also well-connected and/or proximal with the disease-related module in the network. Such a circumstance may arise when nearly all the samples used in network learning for a particular disease were on treatment at the time their blood was sampled. Similarly, some treatments (e.g., metabolite supplementation, enzyme replacement therapy) have shown to successfully normalize disease-relevant metabolite perturbations in targeted, disease pathways. Network models trained on metabolomic profiles without disease-relevant perturbations will not be informative. On the other hand, some treatment-related signatures—especially when the treatment is highly specific to a particular IEM—can improve diagnostic accuracy in some circumstances, as these signatures can provide indirect evidence for the presence of disease.

In summary, our work suggests that data-derived network models offer competitive diagnostic accuracy compared to rule-based biomarker modeling approaches, show improved performance as more data accumulates, and can replace pathway-based modeling approaches, particularly when the relevant pathway knowledge is unavailable.

## Conclusions

The benefit of CTD-based metrics can be particularly powerful when applied to individuals who are undiagnosed by current methods. By quantifying the likeness of individuals’ metabolite perturbations with perturbation patterns observed in many diseases, candidate diagnoses can be ranked and possible diagnoses can be recommended. Furthermore, if genetic sequencing data are available for an individual exhibiting strong disease-specific metabolite perturbation patterns, VUSs can be re-interpreted given the functional evidence provided by untargeted metabolomics. While CTD-based metrics cannot eliminate manual review entirely, they can expedite it and increase the confidence by which clinical laboratory directors make diagnostic decisions. Finally, disease-specific network models can be automatically and continuously updated as new case profiling data accumulates, ensuring stronger network stability and improved diagnostic performance.

## Supplementary Information

Supplementary Information 1.

Supplementary Information 2.

Supplementary Information 3.

## Data Availability

Datasets related to this article are available within Supplemental Table 2 and accessible via the Metabolomics Data Portal Download Data tab (Figure S4).
