# Supplementary material for: Clinical diagnosis of metabolic disorders using untargeted metabolomic profiling and disease-specific networks learned from profiling data
Source: Sci Rep. 2022 Apr 21;12:6556. doi: 10.1038/s41598-022-10415-5 (PMC9023513; doi:10.1038/s41598-022-10415-5)
Supplement: Supplementary file 1 — Supplementary Information 1. [file 41598_2022_10415_MOESM1_ESM.docx]

**Supplemental Information for**

**Clinical Diagnosis of Metabolic Disorders Using Untargeted Metabolomic Profiling and Disease-Specific Networks Learned from Profiling Data**

Lillian R. Thistlethwaite^1,2,*^ PhD, Xiqi Li^2,*^ MS MD, Lindsay C. Burrage^2,5^ MD PhD, Kevin Riehle^2^ MS, Joseph G. Hacia^3^ PhD, Nancy Braverman^4^ MD M.Sc., Michael F. Wangler^2,5^ MD, Marcus J. Miller^6^ PhD, Sarah H. Elsea^2^ PhD, Aleksandar Milosavljevic^1,2,**^ PhD

^1^Quantitative & Computational Biosciences Program, Baylor College of Medicine, Houston, TX

^2^Department of Molecular and Human Genetics, Baylor College of Medicine, Houston, TX

^3^Department of Biochemistry and Molecular Medicine, Keck School of Medicine of the University of Southern California, Los Angeles, CA

^4^Department of Pediatrics and Human Genetics, McGill University, Montreal, Quebec, Canada

^5^Texas Children’s Hospital, Houston, TX

^6^Department of Medical and Molecular Genetics, Indiana University School of Medicine, Indianapolis, IN

*LRT and XL contributed equally to this work

**Corresponding Author:

Aleksandar Milosavljevic, Ph.D.

Department of Molecular and Human Genetics

Quantitative and Computational Biosciences Program

Baylor College of Medicine

One Baylor Plaza, 400D

**SUPPLMENTAL TEXT**

**Supplemental Materials and Methods**

## **Metabolomics analysis and data processing**

For newly acquired samples in this study, metabolomic profiling (Global MAPS®) was performed by Baylor Genetics in collaboration with Metabolon, Inc. (Durham, NC) (www.metabolon.com), as described previously (1, 2). The chemical structures of known metabolites were identified by matching the ions’ chromatographic retention index, nominal mass, and mass spectral fragmentation signatures with reference library entries created from authentic standard metabolites under the identical analytical procedure as the experimental samples (3). Raw spectral intensity values were measured using the area under the chromatographic peak and normalized to the anchor samples, log transformed, and compared with a normal reference population to generate z-scores.

## **Using CTD to determine the main disease module in a disease-specific network.**

While there are multiple ways to define a main disease module, we use the approach outlined in Algorithm 1 below. First, we identify the set of perturbations for all known disease cases which are best explained by the connectedness patterns in the appropriate disease-specific network. Second, we combine the highly connected perturbations across all representative disease cases, and then identify the most connected set of the combined set of perturbations in the disease-specific network.

**Algorithm 1*.* An algorithm to identify the main disease module in a disease-specific network.** The algorithm first finds highly connected metabolite sets within the perturbed metabolites for all individual disease cases. Next, out of the combined set of these highly connected modules, the metabolites found in at least half of all cases were used as input nodes for the CTD method to determine the most connected subset. This subset of metabolites is defined as the main disease module.

| **Algorithm to Identify the Main Disease Module** |
| --- |
| **input :** G, caseIDs, adj_mat |
| G [hash] – node names are KEYS, node probabilities are VALUES |
| caseIDs [vector] – sample IDs from diseased patients |
| adj_mat [matrix] – the weighted adjacency matrix of the disease-specific network |
| **output :** main_dis_mod [vector] – node names of members of the main disease module |
|  |
| 1 disease_mod = [] |
| 2 **foreach** ptID **in** caseIDs: |
| 3 S = get metabolites with z-score < -2 or > 2 for patient ptID |
| 4 *# Use CTD to define mets* |
| 5 mets = highly connected metabolites found in node set S |
| 6 disease_mod = [disease_mod, mets] |
| **7 end** |
| 8 disease_mod = unique(disease_mod) |
| 9 *# Use CTD to define main_dis_mod* |
| 10 main_dis_mod = highly connected metabolites found in node set disease_mod |
| 11 **return** main_dis_mod |

**The Jaccard distance, incorporating information about the directionality of metabolite perturbations.** For some disorders of metabolism, diagnosis between two disorders will depend on whether a given metabolite biomarker is either positively perturbed (i.e., a z-score > 0) or negatively perturbed (i.e., a z-score < 0). Since the CTDdm metric does not take into consideration the directionality of the perturbation observed, we compare the accuracy of the CTDdm distance metric to another approximation of mutual information, the Jaccard distance, which incorporates directionality. We do this by doubling the number of metabolite outcomes in our set space, where each metabolite now has a “+” and a “-” representative member to represent either a positive or a negative perturbation. We distinguish positive and negative perturbations by treating them as different set members (e.g., S_1_ = {arginine+, ornithine-, urea-} and S_2_ = {arginine-, ornithine-, urea-}) and calculate the Jaccard distance accordingly (**Equation 2**).

$Jac\left( S_{1}, S_{2} \right)= 1-\left| S_{1}\cap S_{2} \right|/\left| S_{1}{\cup S}_{2} \right|$ (4)

**Supplemental Results**

## **Combining information from CTD, CTDdm and the Jaccard distance can help identify and correct false positive calls made by CTD alone.**

Metabolomic profiling provides information not only about the consequences of pathogenic variants but also helps monitor patient-specific responses to diet, medications, and other therapeutic interventions (5). Because clinical metabolomics data are inherently confounded by treatment signatures and co-morbid disease signatures, it can be difficult to prune all confounding signatures in the pruning stage of network construction (4). This leaves the possibility that some highly connected node sets in the pruned disease interaction network can be primarily explained by off target disease effects, such as medication signatures or co-morbid disease states. These non-disease-specific signatures can cause false positive CTD calls. CTDdm works by comparing two sets of perturbations in the context of a disease-specific network: the closer two sets of perturbations are in a network context, the more similar those sets of perturbations are with each other. Looking at the distance between patient-specific perturbation modules from a main disease module using CTDdm can help eliminate those false positives calls made by CTD and improve overall diagnostic accuracy.

In **Table S1**, we measured the diagnostic accuracy of CTD, the accuracy of the combined network score (CTD+CTDdm), and the accuracy of the combined network and Jaccard distance score (CTD+CTDdm+Jaccard) separately. First, we quantified the connectedness of the top 30 perturbed metabolites per sample by running CTD on 188 test patient samples with genetic disorders originating from 16 different IEM disorders and 172 “negative control” samples. For several disease states, results were highly specific, with the correct disease showing the strongest signal when profiles were interpreted by the correct disease network, and little to no signal when interpreted by the incorrect disease network. We also calculated the distance between patients’ metabolite perturbations and the main disease module (**Algorithm 1**) in disease-specific networks, using both CTDdm and Jaccard. In order to combine information between CTD and both distance metrics, CTDdm and Jaccard distances were first independently converted into empirical p-values based on the percentile for each distance observed across all patients tested. Empirical p-values for both CTDdm and Jaccard were then combined with CTD p-values using Brown’s combined p-value. We found that combining information from CTD, CTDdm and Jaccard often led to improved diagnostic accuracy compared to the accuracy of CTD alone (**Table S1**).

**The Metabolomics Data Portal**

We created an R shiny application for users to explore the data used in this paper (<https://genboree.org/genboreeKB/projects/metabolomics-data-portal>), from multiple perspectives. The application features four different tabs: View Patient Report (**Figure S1**), Network-Assisted Diagnostics (**Figure S2**), Inspect Reference Population (**Figure S3**) and Download Data (**Figure S4**).

In the View Patient Report tab (**Figure S1**), individual patients’ metabolomics profiles or a mean profile comprised of averaging perturbations observed across several patients can be inspected and visualized. Data can be inspected manually, analyzed by two pathway enrichment methods (e.g., over-representation analysis and metabolite set enrichment analysis), or visualized onto over 50 pathway maps curated by Metabolon (MetaboLync Pathway Visualizations software, version 1.1.2, copyright 2014 Metabolon, Inc., Research Triangle Park, NC, USA).

In the Network-Assisted Diagnostic tab (**Figure S2**), individual patient metabolomics data are interpreted using CTD, CTDdm, or a combined (i.e., CTD+CTDdm) network score, and the evidence from the metabolome is displayed for all patients in a selected disease cohort in all disease-specific network contexts at a given time, in matrix form (**Figure S2b**). We also show a visualization of the patient’s modular perturbations in the disease-network of interest (**Figure S2d**), where one of three network scopes may be selected (e.g., Top K metabolites only, Abnormal metabolites only, and All Metabolites). Of note, we do not recommend the “All Metabolites” option, because it takes a lot of RAM to render; however, it provides the largest scope for the visualization of a given patient’s metabolite perturbations compared to the main disease module. Patient perturbations are connected using purple edges and the metabolites in the main disease module are connected with green edges. When there is overlap between the patient’s highly connected metabolite perturbations and the main disease module, yellow edges connect relevant metabolites. Therefore, the more similar a patient’s perturbations are with a given disease state, the more yellow edges you will see.

In the Inspect Reference Population tab (**Figure S3**), data from “healthy control” reference samples can be inspected at the individual metabolite level. Data can be visualized with histogram plots, Q-Q plots, outlier detection reports and by characterizing the rarity of observing perturbations above clinical thresholds (z-score >2.0 or <-2.0) in “healthy control” reference individuals. Finally, a final tab allows users to download the data used in this paper (**Figure S4**).

**Recommended quantity of profiling data for CTD-based diagnosis.**

Specifying the amount of profiling data required to ensure the quality of CTD-based metrics is of importance in clinical settings, especially for diagnosis of rare diseases, where few numbers of individuals in the world have the disease. To this end, we developed a scoring function to evaluate the stability of network models learned from different numbers of metabolomics profiling samples.

For each disease cohort with n disease profiles, we learned n “network folds”, G_1_ to G_n_, each constructed with one sample excluded from training set **(Table S2)**. Then for each disease, we evaluated the similarity amongst all network folds. The more similar these network folds were, the more stable we considered them. The scoring function we devised for network stability is calculated in two steps. First, for each pair of disease-specific “network folds”, similarity is estimated as the fraction of shared edges out of the union set of the edges from the two graphs. Importantly, an edge is considered shared between two graphs only if the directionality of the edge weights is the same. Second, an overall “network stability” score is determined as the mean of similarity of all pairwise graph comparisons.

Despite the fact that negative control samples are easier to accumulate compared to diseased cases, the number of negative controls required is also of concern for research designs. To address this question, “network folds” were also learned for different sizes of negative control samples, spanning from 15 to 65 samples. While the impact of the number of disease profiles used in network learning on model stability is evaluated by comparing all 16 IEM disease-specific network models, the number of negative control samples on network stability is evaluated only in two diseases, argininemia and argininosuccinic aciduria. Each fold is learned from contrasting all diseased patients against a subset of negative controls randomly selected from a pool of 399 healthy references (data not shown), totaling to 6 folds per control group size.

As shown in **Figure S5**, while the stability of network models generally increases along with accumulation of training data, across all 16 IEM disease-specific models, stability dropped sharply when cohort size decreased from 5 to 3 **(Figure S5A)**. Considering the low prevalence of IEMs, we therefore recommend including a minimum of 5 disease profiles and thus accepting models with stability score of 0.2. Additionally, as the number of negative controls increased, growth of network stability slowed **(Figure S5B)**. Thus, inclusion of more than 55 negative control samples had limited gain **(Figure S5B)**. Nonetheless, in both argininemia and argininosuccinic aciduria models, 25 negative control samples showed stability scores around or above the acceptable threshold **(Figure S5B)**. Based on these results, we suggest using as many as 55 negative control profiles if available and as many disease profiles as available when constructing a disease-specific model. A minimum of 5 disease profiles and 25 negative controls is recommended.

**References**

1 Miller MJ, Kennedy AD, Eckhart AD, Burrage LC, Wulff JE, Miller LA, et al. Untargeted metabolomic analysis for the clinical screening of inborn errors of metabolism. J Inherit Metab Dis. 2015;38(6):1029-39.

2. Ford L, Kennedy AD, Goodman KD, Pappan KL, Evans AM, Miller LAD, et al. Precision of a clinical metabolomics profiling platform for use in the identification of inborn errors of metabolism. J Appl Lab Med. 2020;5(2):342-56.

3. Dehaven CD, Evans AM, Dai H, Lawton KA. Organization of GC/MS and LC/MS metabolomics data into chemical libraries. J Cheminform. 2010;2(1):9.

4. Thistlethwaite LR, Petrosyan V, Li X, Miller MJ, Elsea SH, Milosavljevic A. ﻿CTD: An information-theoretic algorithm to interpret sets of metabolomic and transcriptomic perturbations in the context of graphical models. PLoS Comput Biol. 2021;17(1): e1008550.

5. Burrage LC, Thistlethwaite L, Stroup BM, et al. Untargeted metabolomic profiling reveals multiple pathway perturbations and new clinical biomarkers in urea cycle disorders. *Genet Med.* 2019;21(9):1977-1986.

6. Wangler MF, Hubert L, Donti TR, Ventura MJ, Miller MJ, Braverman N, et al. A metabolomic map of Zellweger spectrum disorders reveals novel disease biomarkers. Genet Med. 2018;20(10):1274-83.

7. Donti TR, Cappuccio G, Hubert L, Neira J, Atwal PS, Miller MJ, et al. Diagnosis of adenylosuccinate lyase deficiency by metabolomic profiling in plasma reveals a phenotypic spectrum. Mol Genet Metab Rep. 2016;8:61-6.

8. Atwal PS, Donti TR, Cardon AL, Bacino CA, Sun Q, Emrick L, et al. Aromatic L-amino acid decarboxylase deficiency diagnosed by clinical metabolomic profiling of plasma. Mol Genet Metab. 2015;115(2-3):91-4.

9. Kennedy AD, Pappan KL, Donti T, Delgado MR, Shinawi M, Pearson TS, et al. 2-pyrrolidinone and succinimide as clinical screening biomarkers for GABA-transaminase deficiency: Anti-seizure medications impact accurate diagnosis. Front Neurosci. 2019;13:394.

10. Alaimo JT, Glinton KE, Liu N, Xiao J, Yang Y, Sutton VR, et al. Integrated analysis of metabolomic profiling and exome data supplements sequence variant interpretation, classification, and diagnosis. Genet Med. 2020;22(9):1560-6..

11. Pappan KL, Kennedy AD, Magoulas PL, Hanchard NA, Sun Q, Elsea SH. Clinical metabolomics to segregate aromatic amino acid decarboxylase deficiency from drug-induced metabolite elevations. Pediatr Neurol. 2017;75:66-72.

**SUPPLEMENTAL FIGURES & TABLES**


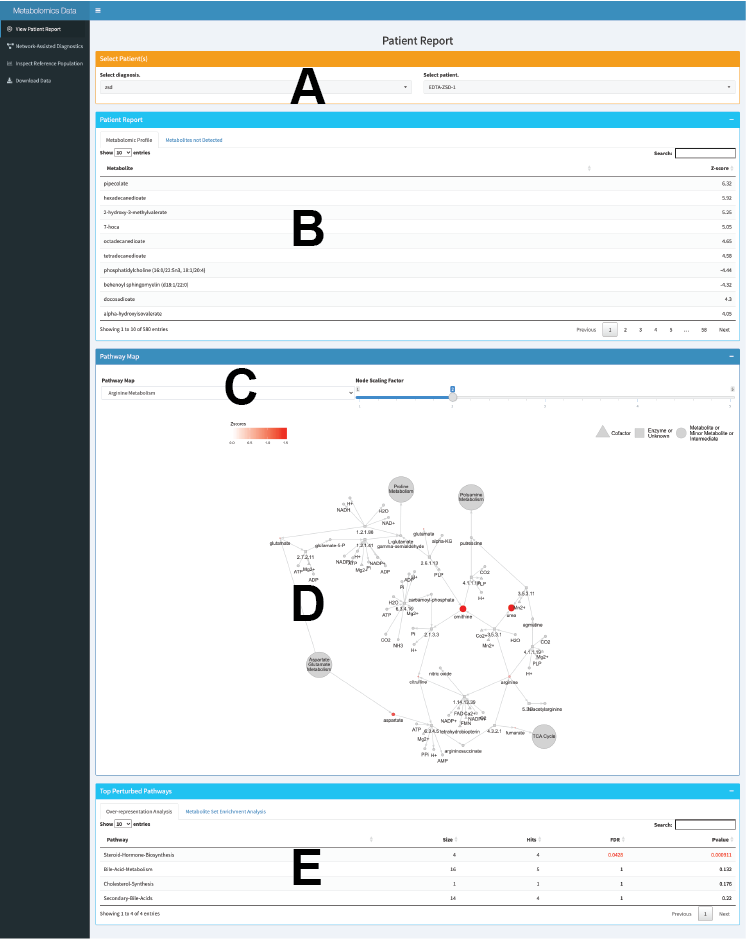


**Supplemental Figure 1. View Patient Report tab.** (A) Select a patient identifier, organized by diagnosis for easier findability. (B) Metabolite z-scores can be viewed, with the strongest perturbations (up or down) listed first. In a second tab, metabolites that are normally detected but were not detected in a given individual are listed, with the percentage of reference samples the compound was detected in. (C) Select the pathway map onto which you want to visualize the selected individual’s metabolite perturbations. Use the scaling factor to resize node labels and modulate the radius of the overlaid perturbations. (D) Z-scored data are overlaid onto pathway maps (Metabolync Pathway Visualizations, v1.1.2). Red circles denote a positive perturbation, whereas blue circles denote a negative perturbation. The radius of the circle denotes the strength of the perturbation. (E) Pathway enrichment results, including over-representation analysis on the individual’s data and metabolite set enrichment analysis for the selected diagnostic cohort.


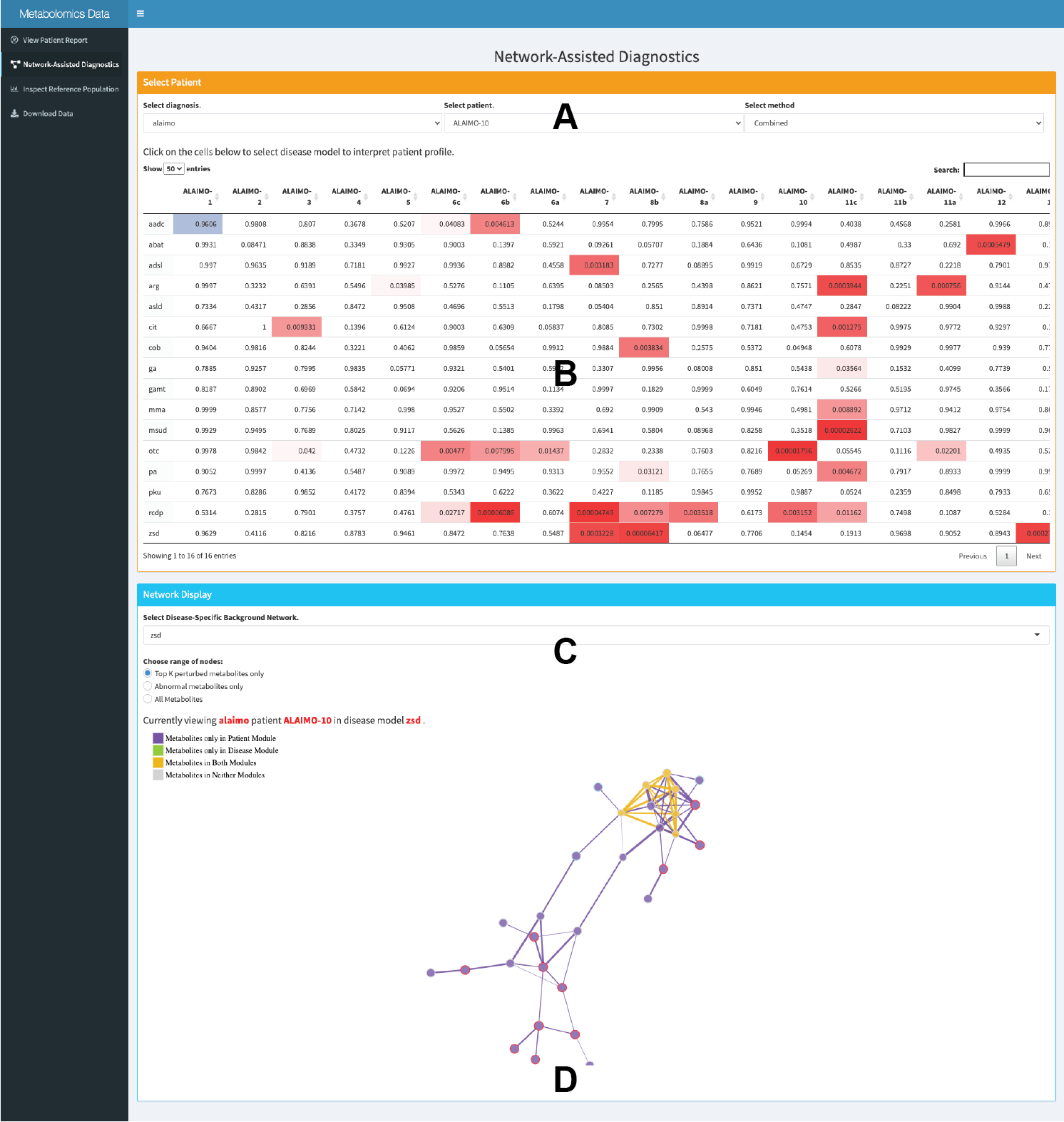


**Supplemental Figure 2. Network-Assisted Diagnostics tab.** (A) Select a patient identifier, organized by diagnosis for easier findability. Also select the metric (e.g., CTD, CTDdm or Combined) you want to see updated in the matrix below. (B) For the selected evidence, p-values are displayed for the selected cohort (columns) across all 16 disease-specific network contexts (rows). Click on any cell to update dropdown values in both (A) and (C). (C) Select the disease-specific network model onto which you want to visualize an individual’s metabolite perturbations. Also select the radio button for the desired network scope. (D) For a given disease-specific network model, you can view the selected patient’s metabolites perturbations (connected in purple) and the disease module (connected in green). When there is overlap between the patient’s metabolite perturbations and the main disease module, yellow edges connect relevant metabolites. Nodes that are perturbed upwards in the selected individual are outlined in red and metabolites that are perturbed downwards are outlined in blue.


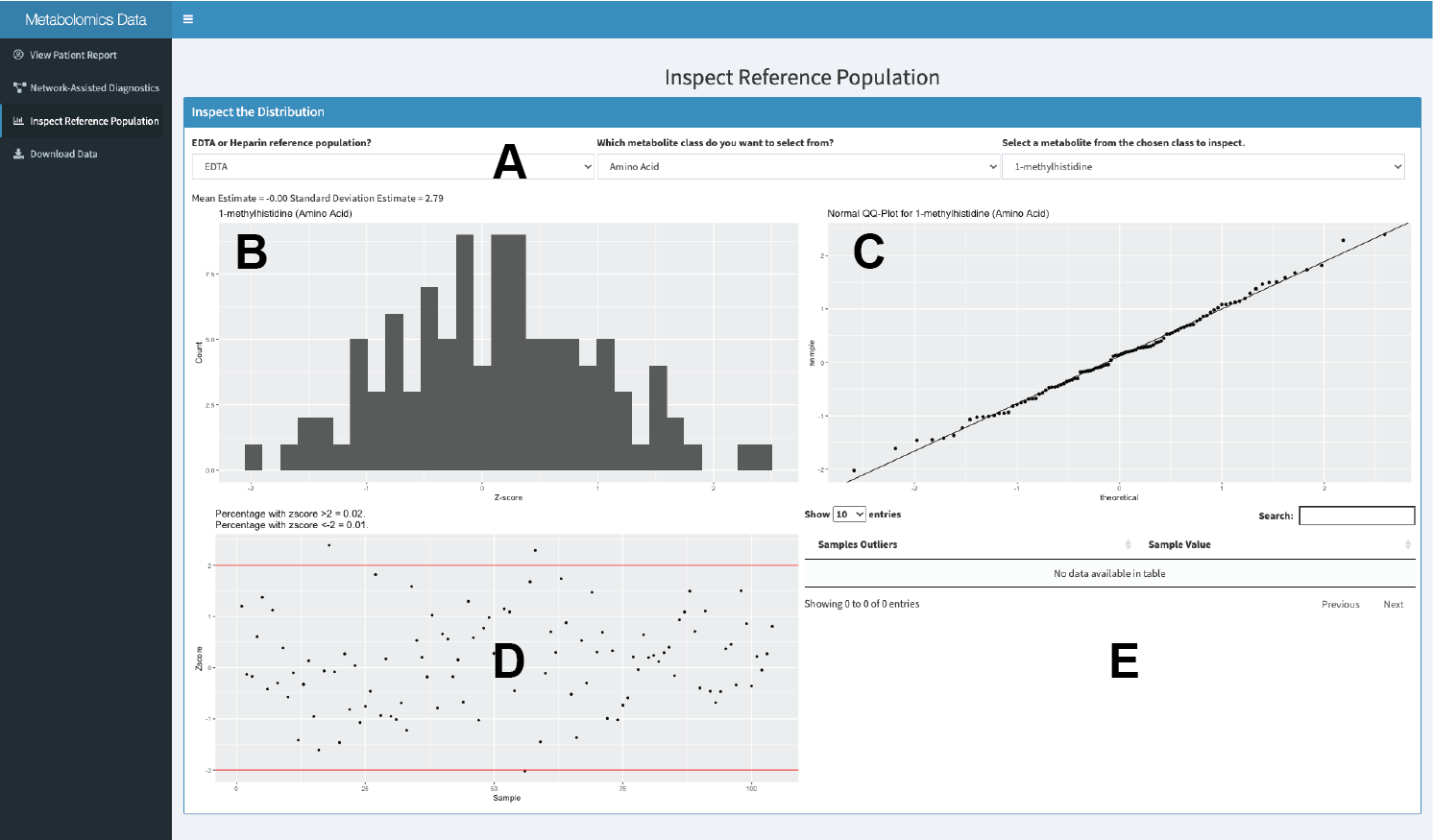


**Supplemental Figure 3. Inspect Reference Population tab.** Reference population samples used in network learning can be inspected by metabolite. (A) Filter by heparin or EDTA-prepared reference samples and select a metabolite of interest first by selecting the compound class, for easier findability. (B) A histogram of the z-scores for the selected metabolite in the selected reference population (e.g., EDTA or heparin) is displayed. (C) A q-q plot for the same data in (A) is shown. (D) A scatter plot illustrating how rare a perturbation above +2.0 or below -2.0 is in the reference population selected. (E) If outlier z-score values are detected, these values are listed in this table. For the metabolite 1-methylhistidine used in this visualization, there were no outliers detected.


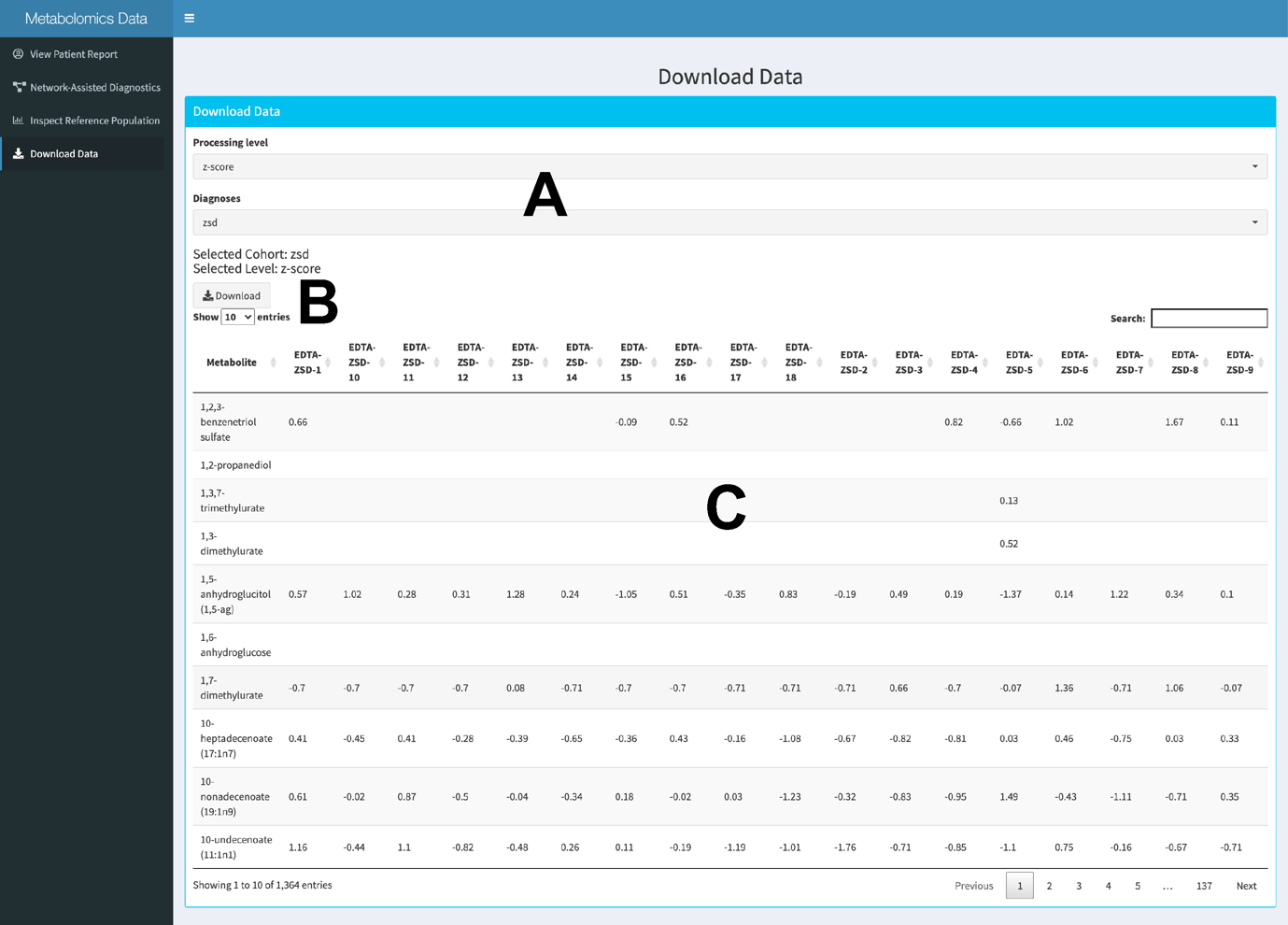


**Supplemental Figure 4. Download Data tab.** All metabolomics profiles analyzed in this paper can be downloaded as a .txt file in this tab. (A) Select processing level as “raw intensity” or “z-scored”, and select one or more diagnoses in the dropdown box. (B) Download the data matrix for the selected individuals by disease state. (C) View the metabolite abundances for the selected cohorts in the matrix.


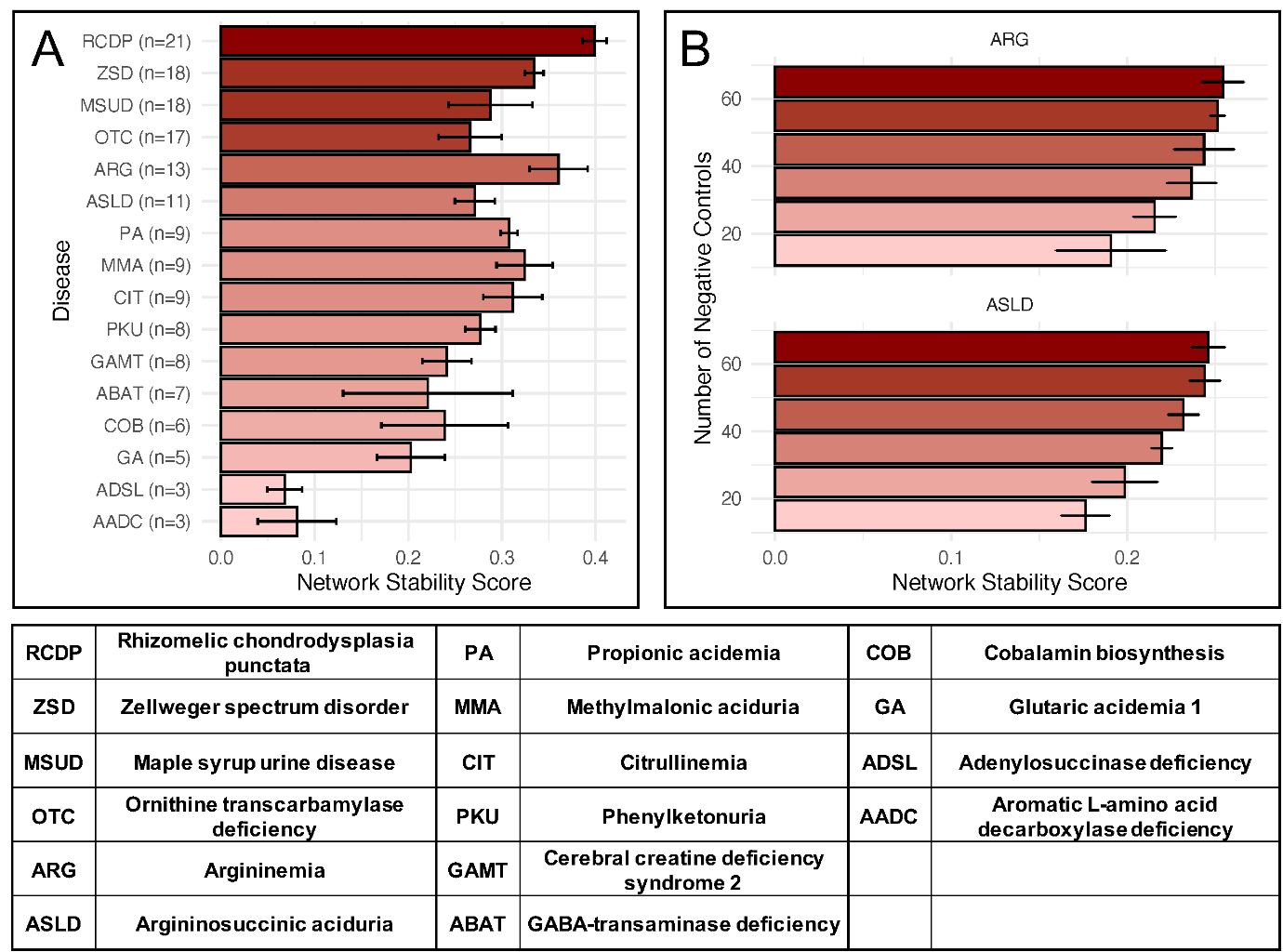


**Supplemental Figure 5. Network stability of disease-specific models with different number of disease profiles (A) and negative controls (B).** Models are ordered and colored in gradient scale by cohort size or size of negative controls. Full names of diseases modeled are noted at the bottom. “Network stability score” is defined as mean of pairwise network folds’ similarity, where similarity is calculated as the fraction of shared edges with the same directionality of edge weights, out of all the edges.

**Supplemental Table 1*.* Information from distance metrics CTDdm and Jaccard when combined with CTD improved diagnostic accuracy.** In the majority of cases, considering i) the relative connectedness of perturbations observed in individual metabolomics profiles in a disease-specific network context (CTD), ii) the distance of those perturbations from the main disease module (CTDdm), as well as iii) information about perturbation membership and directionality (Jaccard) improves diagnostic accuracy compared to considering CTD alone.

| **Diagnosis (OMIM)** | **CTD AUC** | **Combined Network AUC** | **Combined Network+Jaccard AUC** |
| --- | --- | --- | --- |
| Adenylosuccinase deficiency (103050) | 0.485 | 0.802 | 0.951 |
| Argininemia (207800) | 0.981 | 0.985 | 0.989 |
| Argininosuccinic aciduria (207900) | 0.661 | 0.820 | 0.872 |
| Aromatic L-amino acid decarboxylase deficiency (608643) | 0.863 | 0.941 | 0.987 |
| Citrullinemia (215700) | 0.986 | 0.987 | 0.989 |
| Cerebral creatine deficiency syndrome 2 (612736) | 0.944 | 0.951 | 0.948 |
| Cobalamin biosynthesis defect (277400, 277410, 236270, 277380, 250940, 614857, 309541) | 0.938 | 0.965 | 0.968 |
| GABA-transaminase deficiency (613163) | 0.940 | 0.980 | 0.986 |
| Glutaric acidemia 1 (231670) | 0.837 | 0.965 | 0.980 |
| Maple syrup urine disease (248600) | 0.981 | 0.984 | 0.990 |
| Methylmalonic aciduria (251100, 251000) | 0.933 | 0.946 | 0.963 |
| Ornithine transcarbamylase deficiency (311250) | 0.823 | 0.835 | 0.858 |
| Phenylketonuria (261600) | 0.993 | 0.998 | 0.999 |
| Propionic acidemia (606054) | 0.996 | 0.996 | 0.996 |
| Rhizomelic chondrodysplasia punctata (215100) | 0.978 | 0.978 | 0.980 |
| Zellweger spectrum disorder (214100, 601539) | 0.936 | 0.927 | 0.931 |

**Supplemental Table 2. Graphical model metrics for all CTD generated networks.** For each disease cohort with n disease profiles, we learned n “network folds”, each constructed with one sample excluded from training set. Graph density, the ratio of the number of edges |E| with respect to the maximum possible edges.

**File name: Network Model Metrics.xls. Sheet: Disease-specific graph metrics.**

**Supplemental Table 3. Prediction performance of all individual disease models measured by CTD+CTDdm ranks.** For each disease-specific model, a case is predicted positive for this disease when the disease is ranked first by CTD+CTDdm.

| Model | Sensitivity | Specificity | Accuracy (upper bound-lower bound) |
| --- | --- | --- | --- |
| Aromatic L-amino acid decarboxylase deficiency | 1.000 | 0.989 | 0.989(0.962-0.999) |
| GABA-transaminase deficiency | 0.857 | 0.989 | 0.984(0.954-0.997) |
| Adenylosuccinase deficiency | 0.000 | 1.000 | 0.984(0.954-0.997) |
| Argininemia | 0.765 | 0.982 | 0.963(0.925-0.985) |
| Citrullinemia | 1.000 | 0.911 | 0.915(0.865-0.951) |
| Cobalamin biosynthesis | 0.333 | 0.989 | 0.968(0.932-0.988) |
| Glutaric acidemia 1 | 0.800 | 0.984 | 0.979(0.946-0.994) |
| Cerebral creatine deficiency syndrome 2 | 0.750 | 1.000 | 0.989(0.962-0.999) |
| Maple syrup urine disease | 1.000 | 0.971 | 0.973(0.939-0.991) |
| Methylmalonic aciduria | 0.444 | 0.989 | 0.963(0.925-0.985) |
| Propionic acidemia | 1.000 | 0.978 | 0.979(0.946-0.994) |
| Phenylketonuria | 1.000 | 0.989 | 0.989(0.962-0.999) |
| Rhizomelic chondrodysplasia punctata | 0.714 | 0.988 | 0.957(0.918-0.981) |
| Zellweger spectrum disorder | 0.833 | 0.965 | 0.952(0.911-0.978) |
| Argininosuccinic aciduria | 0.462 | 1.000 | 0.963(0.925-0.985) |
| Ornithine transcarbamylase deficiency | 0.441 | 0.961 | 0.867(0.810-0.912) |

**Supplemental Dataset 1. Raw and z-scored metabolomic profiles and patient meta data.** This file includes 1. Z-scored matrices of all 539 samples; 2. raw metabolite intensity of 337 samples from previously reported studies (5, 7-10), as well as previously unreported samples and 3. De-identified treatment records of patients with urea cycle disorders reported by Burrage et al. (5). Raw intensity metrics is not available from Miller et al. (1) and Wangler et al. (6). Note that all previously reported studies were collected in separate batches. Raw data are thus not directly comparable and may contain strong batch-related effects. The z-scored data, however, is similarly processed for all batches to facilitate batch-to-batch comparisons and thus z-scored values can be more directly compared across studies.

**File name: dataset1.xls.**
